# Micrometric pyrite catalyzes abiotic sulfidogenesis from elemental sulfur and hydrogen

**DOI:** 10.1038/s41598-024-66006-z

**Published:** 2024-07-31

**Authors:** Charlotte M. van der Graaf, Javier Sánchez-España, Andrey M. Ilin, Iñaki Yusta, Alfons J. M. Stams, Irene Sánchez-Andrea

**Affiliations:** 1 Laboratory of Microbiology, Wageningen University, Stippeneng 4, 6708 WE Wageningen, The Netherlands; 2 Present Address: Faculty of Civil Engineering and Geoscience, Department of Geoscience and Engineering, Delft University of Technology, Stevinweg 1, 2628CN Delft, The Netherlands; 3 Planetary Geology Research Group, Department of Planetology and Habitability, Centro de Astrobiología (CAB, CSIC-INTA), 28850 Torrejón de Ardoz, Madrid, Spain; 4 Department of Geology, University of the Basque Country (UPV/EHU), Apdo. 644, 48080 Bilbao, Spain; 5 Centre of Biological Engineering, University of Minho, Campus de Gualtar, 4710-057 Braga, Portugal; 6 Present Address: Department of Environmental Sciences for Sustainability, IE University, C. Cardenal Zúñiga, 12, 40003 Segovia, Spain

**Keywords:** Geochemistry, Geochemistry, Element cycles

## Abstract

Hydrogen sulfide (H_2_S) in environments with temperatures below 100 °C is generally assumed to be of microbial origin, while abiotic H_2_S production is typically restricted to higher temperatures (T). In this study, we report an abiotic process for sulfidogenesis through the reduction of elemental sulfur (S^0^) by hydrogen (H_2_), mediated by pyrite (FeS_2_). The process was investigated in detail at pH 4 and 80 °C, but experimental conditions ranged between 40 and 80 °C and pH 4–6. The experiments were conducted with H_2_ as reducing molecule, and µm-sized spherical (but not framboidal) pyrite particles that formed in situ from the H_2_S, S^0^ and Fe^2+^ present in the experiments. Fe monosulfides, likely mackinawite, were identified as potential pyrite precursors. The absence of H_2_ production in controls, combined with geochemical modelling, suggests that pyrite formation occurred through the polysulfide pathway, which is unexpected under acidic conditions. Most spherical aggregates of authigenic pyrite were composed of nanometric, acicular crystals oriented in diverse directions, displaying varying degrees of organization. Although it was initially hypothesized that the catalytic properties were related to the surface structure, commercially sourced, milled pyrite particles (< 50 μm) mediated H_2_S production at comparable rates. This suggests that the catalytic properties of pyrite depend on particle size rather than surface structure, requiring pyrite surfaces to act as electron shuttles between S^0^ and H_2_.

## Introduction

Pyrite (FeS_2_) is the most abundant metal sulfide in the Earth’s crust^1^, and its burial is one of the main sinks of sulfur from the Earth surface^2^. Its sensitivity to oxygen makes it a proxy for past redox conditions on Earth, and the sulfur isotope composition of FeS_2_ is used to assess the contribution of microbial metabolism to its formation^3^. Pyrite formation and its surface chemistry furthermore play a key role in the ‘Iron-Sulfur World’ theory on the origin of life^4^. Pyrite is found in a range of environments and forms under varying temperature (T), pH, and pressure, which together with substrate concentrations and the presence of trace metals influence the formation and morphology of resulting pyrite crystals and aggregates^5–9^. In sulfidic environments, pyrite forms through an aqueous iron monosulfide (FeS_aq_) intermediate (Eq. 1), that is transformed to FeS_2_ either by H_2_S in the sulfide pathway (Berzelius reaction, Eq. 2), releasing H_2_, or attacked by nucleophilic polysulfides (S_n_^2−^) in the polysulfide pathway (Bunsen reaction, Eq. 3), producing FeS_2_ and shorter chain polysulfides. In the latter, elemental sulfur (S^0^) plays a key role by enabling S_n_^2−^ formation through reaction with H_2_S (Eq. 4). A third mechanism for pyrite formation, the ferric-hydroxide-surface pathway, was recently proposed, that becomes relevant when ferric iron concentrations are in excess of H_2_S_aq_^10^.

1 $${Fe}^{2+}+{H}_{2}S\to FeS+2{H}^{+}$$

2 $$FeS+{H}_{2}S\to {FeS}_{2}+{H}_{2}$$

3 $$FeS+{S}_{n}^{2-}\to {FeS}_{2}+{S}_{n-1}^{2-}$$

4 $$\frac{n-1}{8}{S}_{8}^{0} \left(s\right)+{HS}^{-}\to {S}_{n}^{2-}+{H}^{+}$$

In surface environments with T below 100°C, the production of H_2_S (sulfidogenesis) needed for pyrite formation is generally attributed to dissimilatory reduction of oxidized sulfur compounds such as sulfate (SO_4_^2−^), thiosulfate (S_2_O_3_^2−^) or S^011^. Chemical sources of H_2_S were long thought to be relevant only above 100°C. These include disproportionation of sulfur dioxide (SO_2_) to H_2_S and SO_4_^2−^ during volcanic outgassing^2^, and thermochemical reduction of SO_4_^2−^ to H_2_S^12^. Recent work indicated, however, that this boundary is not as clear. Pyrite, long considered unreactive under anoxic conditions and ambient temperatures, was shown to undergo reductive dissolution by H_2_ at elevated H_2_ pressures (8 bar) and alkaline pH (> 7) at 90 °C, producing pyrrhotite and H_2_S^13^. Reductive dissolution of pyrite was observed even at T as low as 37 °C, although this was not strictly abiotic, as it involved the activity of methanogenic archaea^14,15^.

In this study, we describe a novel abiotic route for sulfidogenesis at acidothermal conditions involving pyrite. Instead of H_2_S originating from its reductive dissolution, pyrite is proposed to act as a catalyst for electron transfer from H_2_ to S^0^. This abiotic process was discovered in incubations performed at acidothermal (pH 4, 80°C) conditions, which were designed to enrich S^0^-reducing thermoacidophilic microorganisms from acidic volcanic hot pools. The abiotic process was studied under varying conditions, with controls, to assess the potential role of different chemical compounds as well as the influence of pH and temperature. The mineral precipitates that formed during the process were characterized by a range of mineralogical techniques, confirming the formation of pyrite. We furthermore showed that pyrite by itself enabled the reduction of S^0^ by H_2_. Although pyrite is known to have catalytic properties, its ability to catalyze the reduction of S^0^ with H_2_ has not been described previously and expands our understanding of the geological and biological sulfur cycle in acidothermal environments.

### Pyrite mediates sulfidogenesis from elemental sulfur and hydrogen

In microbial enrichment experiments (pH 4, 80 °C) aiming to select for thermoacidophilic, S^0^-reducing microorganisms we observed sulfidogenesis from S^0^ and hydrogen (H_2_) in the presence of Fe^2+^ as reducing molecule. A chemical nature of the process was initially not contemplated because (1) previous research described chemical H_2_S production only above 100 °C; (2) H_2_S curves in minimal salts (MS) medium supplemented with yeast extract (YE) resembled typical microbial batch growth curves (Supplementary Figure s1A-1), and (3) sulfidogenesis was absent in the (sterile) uninoculated controls within the same timeframe. However, extensive attempts to extract DNA, using cultures of the thermoacidophilic sulfur-reducing archaeal species *Acidianus ambivalens* as positive control, were not successful, and no conclusive images of microbial cells could be obtained through TEM, SEM, and light microscopy (results not shown). It was subsequently recognized that the uninoculated controls lacked an essential component initiating the chemical process: the H_2_S present in the active ‘cultures’ used as inoculum.

**Figure 1:**
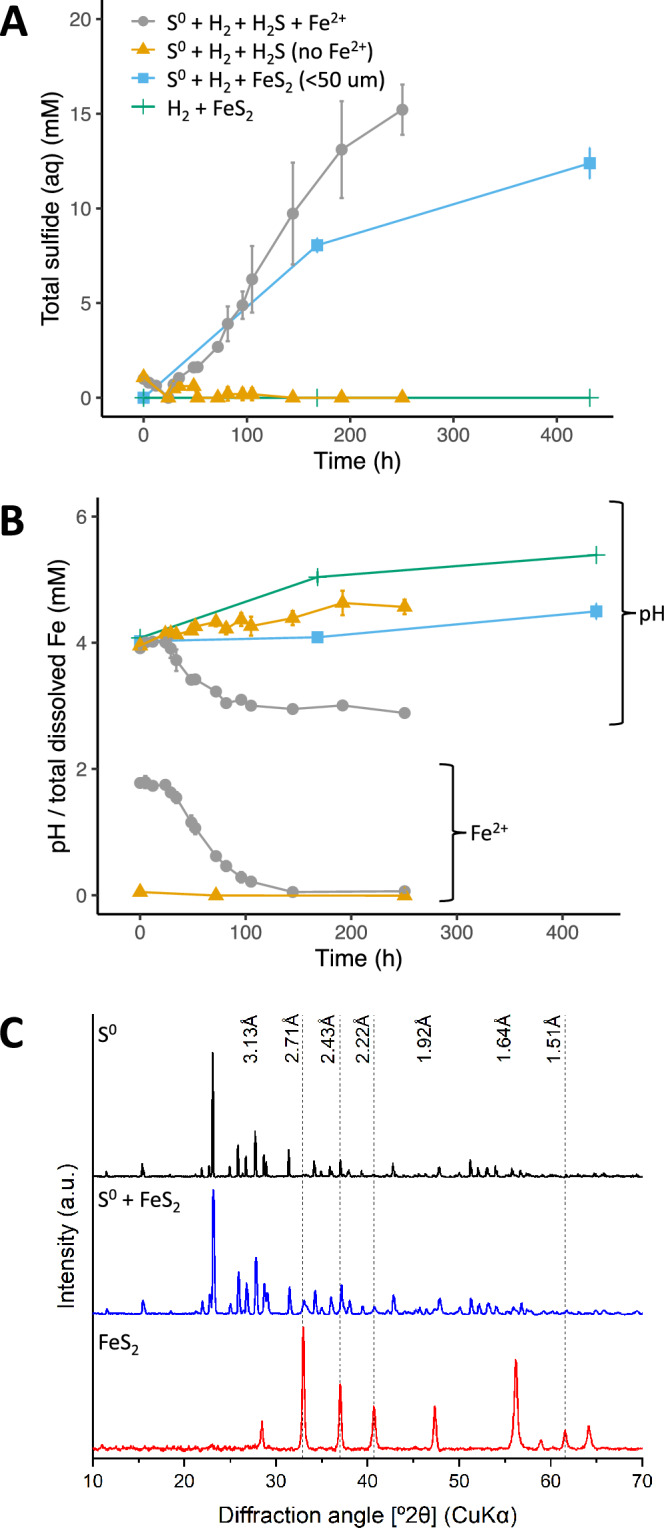
Total sulfide (mM) (**A**), total dissolved iron (mM) and pH (**B**), and XRD analysis of formed precipitates (**C**) in experiments carried out at 80 °C, starting pH 4. A. total sulfide concentrations in the liquid and gas phase expressed over the remaining liquid volume in experiments with mineral salts (MS) medium + S^0^, H_2_, H_2_S supplementation (indicated with H_2_S in the legend) and Fe^2+^ (grey circles); MS medium + S^0^, H_2_, H_2_S supplementation but no Fe^2+^ (orange triangles); deionized water (DI) + S^0^, H_2_, and FeS_2_ (milled and sieved to < 50 µm) (blue squares); or DI + H_2_ and FeS_2_ only (green plus sign). (**B).** pH and dissolved iron concentration in incubations described in A. (**C).** XRD diffractograms for precipitates obtained from experiments with MS medium + YE + S^0^ + Fe^2+^  + H_2_S without H_2_ after 63 days (top), representing the late part of the initiation phase preceding sulfidogenesis or with H_2_ after 5 days (middle) representing the early stage of sulfidogenesis; and after all S^0^ had been converted (day 28) (bottom). The mention of S^0^ and FeS_2_ on the left part of the graphs indicates which minerals were identified in the XRD diffractograms.

To test the hypothesis of chemical sulfidogenesis occurring under these conditions, the process was investigated in detail in sterile experiments at pH 4 and 80 °C (Fig. 1A, B). The process was also found to occur at lower temperatures (pH 4, 60 °C), or higher pH values (6, 80 °C), but not at an initial pH of 2 at 80 °C (Supplementary Figure s2). Interestingly, the presence of YE (a component of the original “microbial enrichment” experiments) was found to extend the initiation phase preceding the onset of sulfidogenesis (Supplementary Figure s1A-1). The formation of a fine black precipitate in the abiotic H_2_S-producing experiments, which did not disappear after all S^0^ had been reduced, combined with the requirement for Fe^2+^, intended as a reducing agent in the original anaerobic microbial enrichments, suggested the involvement of iron sulfides. This was further supported by the observed drop in pH during incubation (Fig. 1B), as formation of FeS from Fe^2+^ and H_2_S releases protons (Eq. 1). X-ray diffraction (XRD) analysis of precipitates harvested after 63 days from the experiments without H_2_ (MS medium + YE + S^0^), detected S^0^ as the only crystalline phase (Fig. 1C, top). Solids from early sulfidogenesis (MS medium + YE + S^0^ + H_2_, H_2_S_tot_ = 3.0 mM, 5 days) contained both pyrite (FeS_2_) and S^0^ (Fig. 1C, middle), while precipitates harvested 16 days after sulfidogenesis ceased (MS medium + YE + S^0^ + H_2_, day 28, H_2_S_tot_ = 20.3 ± 1.6 mM), identified pyrite as the only crystalline phase (Fig. 1C, bottom). The key role of pyrite in chemical sulfidogenesis from S^0^ and H_2_ was confirmed by the observation that supplementation of commercially sourced, milled (< 50 μm, 2 mM) pyrite, instead of pyrite formed in situ from Fe^2+^ and S^0^, also enabled chemical sulfidogenesis, although at a comparatively lower rate (Fig. 1A). In these experiments, pyrite was added to a concentration of 2 mM, based on the amount that could have formed in the experiments from the supplemented Fe^2+^ (2 mM).

Total H_2_S production rates in experiments where pyrite formed in situ or was added externally (Fig. 1A) indicated that this occurred at 1.9 ± 0.5 mM ⋅ day^−1^ or 1.2 mM ⋅ day^−1^, respectively, although in the case of milled pyrite this was only based on two timepoints. Comparison with estimated sulfide production rates for e.g. the thermoacidophilic sulfur-reducing archaeal species *Acidianus* DS80 grown with H_2_ and S^0^ at pH 3.0 and 80 °C (approximately 0.5 mM ⋅ day^−1^)^16^ suggest that abiotic and biological sulfur reduction rates at these conditions could be in a similar range.

Although abiotic sulfidogenesis from S^0^ at elevated temperatures (88 – 110 °C) was reported previously^17^, this was ascribed to S^0^ disproportionation, as it did not require a reducing molecule such as H_2_, and H_2_S concentrations only reached about 0.01 mM after 24 h. However, in the experiments reported here, H_2_S concentrations were almost 1000 times higher, reaching 7–8 mM in the liquid phase, H_2_ was required, and no increase in sulfate (SO_4_^2−^) concentrations (byproduct of disproportionation) was observed (Supplementary Figure s3). This, together with the unfavorable Gibbs free energy change for sulfur disproportionation even at low H_2_S concentrations^18^, ruled out chemical disproportionation as the origin of sulfidogenesis in these experiments. Instead, the requirement for pyrite, either through in situ formation or added from an external source, as well as the requirement for H_2_ (Fig. 1A), suggested that sulfidogenesis resulted from the reduction of S^0^ by H_2_ and was mediated by pyrite, a process that has not been previously described.

Two possible mechanisms for chemical sulfidogenesis involving pyrite were considered: (i) the reductive dissolution of pyrite by H_2_ to H_2_S and a non-stoichiometric iron monosulfide (Eq. 5) or Fe^2+^ (Eq. 6)^13,22^; or (ii) electron transfer from H_2_ to S^0^ enabled by the conductive properties of pyrite (Eq. 7)^19,20^.

5 $${\text{FeS}}_{2}+\left(1-\text{x}\right){\text{ H}}_{2}\to {\text{FeS}}_{1+\text{x}}+\left(1-\text{x}\right){\text{ H}}_{2}\text{S}$$

6 $${\text{FeS}}_{2}+{\text{H}}_{2}+2{\text{H}}^{+}\to {\text{Fe}}^{2+}+2{\text{H}}_{2}\text{S}$$

7 $${\text{H}}_{2}+\frac{1}{8}{\text{S}}_{8}^{0}\stackrel{{\text{FeS}}_{2}}{\to }{\text{H}}_{2}\text{S}$$

The first possibility, reductive dissolution of pyrite by H_2_, would produce H_2_S and either Fe monosulfide (Eq. 5) or Fe^2+^ (Eq. 6), enabling regeneration of FeS_2_ from Fe^2+^ and S^0^ (Eqs. 1–3), maintaining a ‘cycle’ until all S^0^ is consumed. Reductive dissolution of pyrite by H_2_ has been reported at elevated H_2_ partial pressures (> 8 bar, or [H_2_ (aq)] > 7 mM) and elevated temperature (T > 90 °C), at nuclear waste disposal sites^13,21^ and underground H_2_ storage facilities^22^, but also at milder conditions (38 °C, H_2_ (aq) = 0.2 mM) in microbial incubations with methanogenic archaea^14,15^. Although these studies showed that extreme temperatures and H_2_ partial pressures are not necessary for reduction of pyrite by H_2_, they involved neutral to alkaline pH values^13–15^, not the acidic pH values used in the current study. Furthermore, the absence of H_2_S production in the experiments with only pyrite and H_2_ (without S^0^) (Fig. 1A), indicates that reductive dissolution of pyrite did not occur.

The second mechanism, proposing pyrite as a catalyst, is in line with recent observations that in contrast to bulk pyrite, nm- to μm-sized pyrite crystals display conductive properties^19,20,23,24^. For example, they have been proposed to (1) confer electrical conductivity in hydrothermal vent chimneys^25–27^, (2) act as conductors in microbial extracellular electron transfer^28^, and (3) serve as more sustainable electrocatalysts for e.g. photovoltaic cells and water electrolyzers^20^. According to this second hypothesis, the pyrite spheroids would catalyze the oxidation of H_2_ to 2H^+^, and facilitate the subsequent transfer of 2 e^−^ to oxidized sulfur species such as S^0^ or polysulfides, resulting in H_2_S formation. This hypothesis is strongly supported by the observation of sulfidogenesis with externally sourced pyrite in the presence of H_2_ and S^0^ (Fig. 1A), while no H_2_S increase was observed when S^0^ was absent. This indicates that H_2_ does not directly react with the sulfur atoms in the FeS_2_ at the conditions used in our study, but instead solely requires the presence of pyrite as a catalyst.

### Formation of spherical pyrite aggregates composed of lenticular nanocrystals under acidic conditions

**Figure 2:**
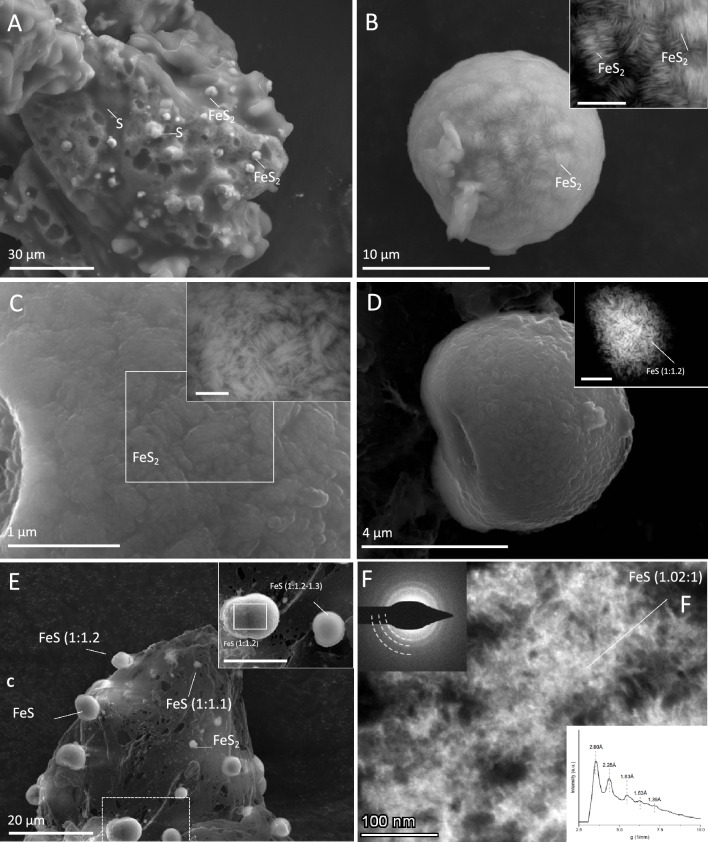
SEM (**A**-**E**) and S/TEM (**F**) micrographs showing morphology, surface structure and Fe:S ratios of iron sulfides formed in the experiments with MS medium + S^0^ + Fe^2+^  + H_2_ + H_2_S, and without yeast extract (YE) (**A**, **B**) or with YE (**C**-**E**). A. pyrite (FeS_2_) and sulfur (S) particles detected on larger sulfur grain in experiments without YE) after 1 day. B. Spherical pyrite particle from an experiment without YE after 4 days, inset: magnified backscatter electron (BSE) image, scalebar 2 µm. C. Surface structure of an FeS_2_ particle from an experiment with YE after 8 days, inset: BSE image, scalebar 0.5 µm. D. Iron monosulfide, inset: BSE of the same particle, scalebar 1 µm. E. iron monosulfides and FeS_2_ detected on sulfur grain after 8 days. Inset: enlarged image of area indicated by the dotted line, scalebar 9 µm. F. Aggregate of acicular nanocrystals of mackinawite shown by HAADF STEM. Left insets: SAED pattern and intensity versus distance (g: 1/nm) profile. Atomic % measured by EDX are shown in Supplementary File 1.

The morphology and internal structure of the precipitates formed in the experiments, and the presence of other low-crystalline iron sulfides in these precipitates was investigated with Scanning Electron Microscopy (SEM) and Scanning Transmission Electron Microscopy (S/TEM). While not detected by XRD (Fig. 1C), SEM coupled to energy dispersive X-ray (EDX) analysis of samples from the later stage of the initiation phase preceding the onset of sulfidogenesis showed neoformed pyrite growing on larger S^0^ particles, indicating that pyrite was already present before the onset of sulfidogenesis (Fig. 2A). Furthermore, pyrite particles were detected in samples harvested after 192 h from a control experiment with MS medium + YE, plus H_2_S and Fe^2+^ but without H_2_, representing the later stage of the initiation phase preceding sulfidogenesis (Supplementary Figure s4). The pyrite particles observed in the different samples were predominantly spheroids, composed of lenticular or acicular nanocrystals displaying varying degrees of organization, with the most ordered particles showing patches of crystals aligned lengthwise in the same direction and resembling a woolball-type structure (Fig. 2B). Visual inspection indicated that the presence of YE could play a role in the size and organization of the FeS_2_. In the absence of YE, a higher degree of organization was observed at an early stage of sulfidogenesis ([H_2_S_aq_] = 0.3 mM, pH 3.5) (Fig. 2B), compared to the experiments with YE ([H_2_S_aq_] = 1.2 mM, pH 3.8) (Fig. 2C). This potentially slowing effect of YE on the process of crystal growth and aggregation is consistent with the previously observed inhibitory effects of organic compounds on pyrite formation^29–31^.

EDX analysis on several iron sulfide particles from experiments with MS medium + YE harvested after 192 h, showed an Fe:S atomic ratio of 1:1.1 – 1:1.3, suggestive of sulfur-rich iron monosulfides (FeS) (Fig. 2D, E). These FeS particles were far more difficult to detect than pyrite particles, and could not be found in all samples, suggesting low abundance, high reactivity, or both. Upon closer inspection, lenticular or rod-like nanocrystals were observed on the surface of some FeS particles, resembling those seen on the pyrite spheroids, as well as those found in synthetic mackinawite^32^, but without a discernible organization (Fig. 2D). Several spherical particles were identified consisting of only sulfur, indicating that S^0^ was also present as globular particles (Fig. 2A), possibly due to the decomposition of polysulfides to nanocrystalline sulfur^33^.

Given the low crystallinity of the precipitates and the abundance of S^0^ masking other phases in XRD diffractograms of precipitates sampled preceding the onset of sulfidogenesis (48 h), S/TEM in brightfield (BF), high-angle annular dark-field (HAADF), and selected area electron diffraction (SAED) modes coupled to EDX analysis was used to investigate the mineralogical evolution of the system in more detail. This showed a change in crystallinity from the early amorphous precipitates towards acicular nanocrystals in highly porous aggregates and their subsequent agglomeration (Fig. 2F). S/TEM–EDX analyses showed two categories of particles, with Fe:S atomic ratios of 1–1.2:1 and 0.44–0.5:1, consistent with the presence of Fe monosulfide and their evolution into Fe disulfides as shown by SEM–EDX. SAED showed either no diffraction (not shown), indicating an amorphous Fe sulfide precipitate, or a ring pattern, indicating polycrystalline aggregates of randomly oriented discrete nanocrystals, each producing diffraction spots distributed at a certain distance from the transmitted beam, indicative of d-spacing of the mineral^34^. These interplanar distances and chemical composition are consistent with mackinawite (tolerance ± 0.10 Å; left inset, Fig. 2F; Supplementary Figure s5A-C) and pyrite (tolerance ± 0.05 Å; Supplementary Figure s5D-L). The loss of continuity of the rings in the pyrite pattern could suggest the coarsening of the individual nanocrystals, compared to mackinawite.

The detection of iron monosulfides agrees with the two main mechanisms proposed for pyrite formation described above (Eq. 1–4), as both the polysulfide and the sulfide pathway^1^, involve an (aqueous) FeS intermediate (Eq. 1). Furthermore, mackinawite is a common product of the reaction of Fe(II) and S(-II) in aqueous solutions, and is considered a common intermediate product in the formation of pyrite^35^. Both pathways are thermodynamically favorable at 85 °C (358 K, the tabulated temperature for Gibbs energy of formation^36^), and the reactant concentrations present at the start of the experiments (0.002 M Fe^2+^, 0.0005 M H_2_S_aq_, 0.0001 M H^+^ (pH 4), 0.0012 mM H_2_ (aq), activity of pyrite and S^0^ of 1), giving − 52.2 kJ/mol for the polysulfide pathway and − 3.5 kJ mol^−1^ for the sulfide pathway. However, this does not indicate whether the kinetics of the two different mechanisms are favorable, as also emphasized previously^1^.

It is generally accepted that at more acidic pH values, the sulfide pathway is the dominant mechanism for pyrite formation^1,6^, largely due to the instability and extremely low concentrations of polysulfide at acidic pH^37,38^. This implies the formation of H_2_ (Eq. 2), which in our experiments would correspond to 0.37 mM H_2_, calculated assuming a complete conversion of approximately 50 μmol of H_2_S added on day 0 to FeS_2_ (Eq. 1 and 2). However, H_2_ did not accumulate after 21 days in our experiments with S^0^, Fe^2+^ and H_2_S (without H_2_) (Supplementary File 2) (the detection limit of our analytical equipment was 3 orders of magnitude lower than 0.37 mM). This suggests that pyrite formation in our experiments occurred through the polysulfide mechanism. However, at this stage, this interpretation is speculative, and it should be noted that previous investigations reported difficulties with recovering H_2_ from systems where pyrite formed through the sulfide pathway due to H_2_ adsorption on pyrite surfaces^39^.

**Figure 3:**
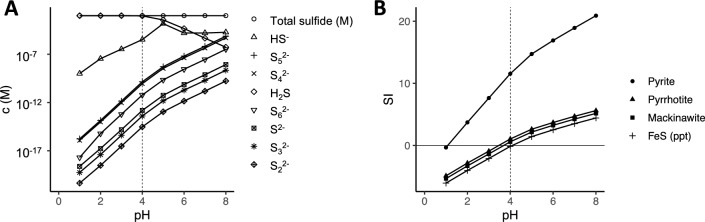
Modeled concentrations of ionic sulfide species (**A**) and saturation indices (SI) of relevant iron sulfides (**B**) for the geochemical conditions prevailing in the experiments (80 °C, 1 mM total sulfide, 1.5 mM Fe^2+^).

The modeled concentrations of polysulfide species such as S_4_^2−^ or S_5_^2−^ in the aqueous solutions of our experiments are on the order of 10^–10^ M (Fig. 3A), consistent with previous research at comparable pH conditions^33^. However, it could be hypothesized that because polysulfides form at the surface of sulfur particles, this enabled higher local polysulfide concentrations than in the bulk liquid^40^, providing locally favorable conditions for pyrite formation through the polysulfide pathway. The occurrence of FeS and FeS_2_ spheroids on the surface of S^0^ particles, combined with possible ‘dissolution pits’ (Fig. 2A), could support an important role for the surface of S^0^ in pyrite nucleation and crystal growth, as also hypothesized in previous studies on the synthesis of hydrothermal pyrite^9,41,42^. Although these studies considered higher temperatures (150 – 350 °C), and the involvement of liquid sulfur droplets, the spherical pyrite particles synthesized in the presence of excess S^0^ were similar to those observed in the current study, and in some cases, also appeared to be embedded in the S^0^ surface^41^.

The spherical shape of the pyrite particles is in agreement with previous work indicating that this occurs at supersaturation of the aqueous solution with respect to pyrite solubility^43^, and under acidic conditions^29^. This was the case in our system, as shown by equilibrium solubility and speciation calculations (Fig. 3B). The size of the pyrite spheroids further indicates that nucleation was favored over growth, which is typical for supersaturation conditions^44^. Although the pyrite spheres resembled the shape and dimension of framboidal pyrite, no clear framboidal inner structure could be determined^42^. Furthermore, it has been proposed that spherical pyrite could be an indicator for (past) biological activity, and recent work suggested that the formation of spherical pyrite particles required the presence of organic matter^45^. However, the results presented in the current study, further emphasize that organic matter is not required for the formation of spherical pyrite particles.

**Figure 4:**
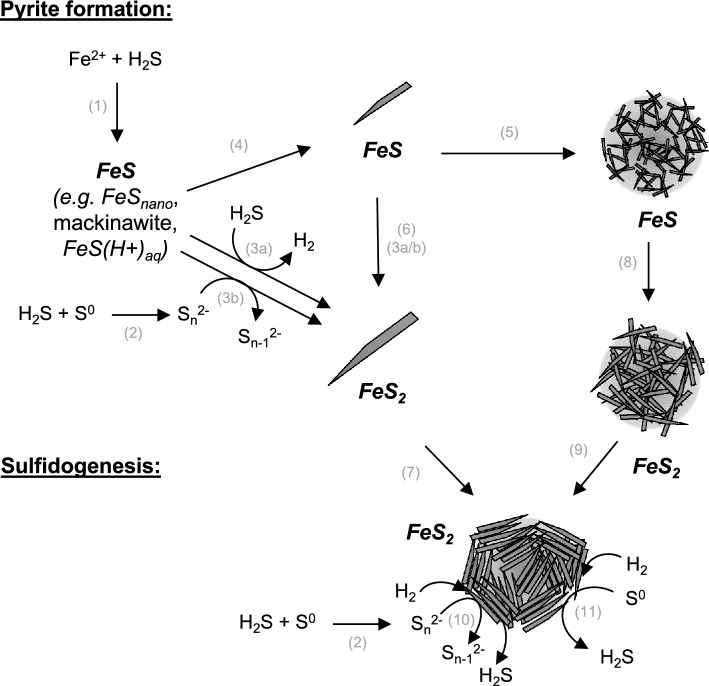
Schematic representation of potential routes for pyrite formation and sulfidogenesis in the current study. (1) formation of FeS clusters or aqueous FeS(H^+^)_aq_ complexes; (2) formation of polysulfides (S_n_^2−^) from H_2_S and S^0^; (3a) pyrite formation from FeS through the sulfide pathway, (3b) pyrite formation through the polysulfide pathway; (4) formation of FeS lenticular nanocrystals; (5) aggregation of FeS lenticular nanocrystals to form FeS spheroids; (6) formation of FeS_2_ from FeS via either the sulfide or polysulfide pathway (see 3a and 3b); (7) organized aggregation of FeS_2_ lenticular crystals into FeS_2_ spheroids; (8) formation of FeS_2_ aggregates from FeS via the sulfide or polysulfide pathway (see 3a and 3b) after FeS aggregation; (9) unknown mechanism for increased organization of FeS_2_ aggregates; sulfidogenesis from H_2_ and (10) (S_n_^2−^) or (11) S_8_^0^ catalyzed by pyrite.

The presence of the Fe monosulfide mackinawite (Fe_1-x_S), as suggested through (S)TEM–EDX, supports the idea that pyrite formation occurred via a solid FeS intermediate, possibly amorphous FeS, or mackinawite, or a recently described ‘novel’ nanocrystalline FeS compound (FeS_nano_)^46^. This FeS_nano_ was stable under acidic pH (< 4.5) and reducing conditions, closely resembling the conditions applied in our study. The identification of the non-stochiometric iron sulfide as mackinawite agrees with the abundant literature on the Fe sulfide precipitation sequence in anoxic environments^32,47,48^. It is possible that mackinawite or FeS_nano_ formed as a solid precursor phase from Fe^2+^ and H_2_S in the experiments, and subsequently reacted with (poly)sulfide to form pyrite. The occurrence of spherical aggregates of acicular FeS crystals could suggest that, at least in some cases, this process occurred after aggregation of nano-crystals into spheroids. However, it cannot be excluded that FeS_2_ formation also occurred prior to aggregation. It could be speculated that the degree of organization of the nanocrystals observed on the spheroid surfaces (Fig. 2) is related to the order of FeS_2_ formation and aggregation. Increased organization could result when aggregation occurs after FeS_2_ formation, and a lower degree of organization while formation of FeS_2_ from FeS takes place after aggregation. Although further research is needed to confirm the exact mechanism, Fig. 4 provides a schematic representation of the proposed steps for pyrite formation.

### Implications

The temperature and pH at which pyrite-catalyzed chemical S^0^ reduction by H_2_ is reported in the present study resemble those found in, for example, acidic hydrothermal vents and acidic geothermal pools. These ecosystems can also have inputs of H_2_S and H_2_ gases from sources such as volcanic sources, and contain significant deposits of S^0^ and nano- to micrometric pyrite particles, as shown for hydrothermal plumes^49,50^ and vent chimneys^25^. The pyrite-type minerals in hydrothermal vent chimney walls were already shown to have conductive properties^25,27^, enabling H_2_ or S^0^ oxidation coupled to O_2_ reduction, but the oxidation of H_2_ coupled to S^0^ reduction in these environments has not been investigated.

Given the similarity of the geochemical conditions, and the observation that increased salinity did not significantly impact the timeframe within which S^0^ was reduced (Supplementary Figure s6), it could be expected that pyrite-catalyzed S^0^ reduction with H_2_ occurs in (deep-sea) hydrothermal environments. Subsequent studies are needed to investigate how the process is affected by the lower H_2_ partial pressures found in natural environments^51,52^. If pyrite-catalyzed S^0^ reduction is indeed found to occur in natural environments, it would nuance the generally held assumption that environmental sulfide production below 100 °C is the result of dissimilatory microbial metabolism of sulfur compounds. Although the sulfur isotopic fractionation introduced by this novel abiotic sulfidogenic pathway is expected to be negligible, further experimental work is needed to evaluate the effects of this S^0^ reduction mechanism on the δ^34^S ratio, and the possible interference with the highly fractionated (strongly negative) biogenic ratios. Finally, our work furthermore underscores that spherical pyrite particles are not a reliable indicator for the presence of organic matter.

## Materials and methods

### Anaerobic batch experiments

Experiments were performed in 117 mL glass serum bottles capped with butyl rubber stoppers (Ochs Laborbedarf, Bovenden, Germany). Anoxic solutions were prepared by boiling and subsequent cooling under continuous sparging with N_2_ gas. Original microbial enrichment screenings were performed with anoxic minimal salts (MS) medium with 0.1 g·L^−1^ yeast extract (YE) (Becton Dickinson, Cockeysville, MA). This medium is referred to as MS medium + YE. Subsequent chemical experiments were performed with either MS medium or acidified demineralized water (DI), with or without YE, at a starting liquid volume of 50 mL. The MS medium composition (in mM) was 2 KH_2_PO_4_, 2.81 (NH_4_)_2_SO_4_, 1.71 NaCl, 0.50 MgSO_4_·7H_2_O, 0.75 CaCl_2_·2H_2_O, and a trace element solution^53^ modified according to the water chemistry of acidothermal volcanic waters^54^, consisting of (in mM): 49.4 HCl, 1.0 H_3_BO_3_, 0.5 MnCl_2_, 7.4 FeCl_2_, 0.5 CoCl_2_, 0.1 NiCl_2_, 0.5 ZnCl_2_, 0.1 CuCl_2_, 10 NaOH, 0.1 Na_2_SeO_3_, 0.1 Na_2_WO_4_, 0.1 Na_2_MoO_4_, 0.1 AlCl_3_, 0.5 RbCl, 0.1 BaCl_2_, 0.1 SrCl_2_, 0.02 VCl_2_, 0.006 PbCl_2_, 0.004 CdCl_2_. Anoxic MS medium was autoclaved without vitamins, CaCl_2,_ YE, and FeCl_2_·4H_2_O. The effect of increased salinity was investigated in acidified demineralized water with 3.0 g·L^−1^ Na_2_SO_4_ and 21.0 g·L^−1^ NaCl (saline DI).

The pH of the liquid was adjusted to 3.8, 2.9, or 1.9 before autoclaving, with 2 M HCl for the original enrichments, and with 1 M–5 M H_2_SO_4_ solutions for the later sterile experiments. pH-adjusted liquid was aliquoted under N_2_/CO_2_ flow to 117 mL serum bottles containing 25 mM of colloidal or orthorhombic chemical elemental sulfur (S^0^) (Sigma Aldrich, St. Louis, MO), and the headspace was exchanged 5 times with N_2_/CO_2_ or H_2_/CO_2_ with an automatic gas exchanger to a final pressure of 1.7 atm (room temperature). Bottles were autoclaved at 105 °C for 30 min. Where applicable, YE, vitamins, and CaCl_2_ were added after autoclaving from sterile stock solutions to their respective final concentrations. Ferrous iron was added as a reducing agent from a sterile anoxic 1 M FeCl_2_.4H_2_O solution to a final concentration of 2 mM. In chemical experiments without Fe^2+^, L-cysteine was added from a pH-adjusted stock solution as reducing agent to a final concentration of 1 mM.

Sulfide was supplemented from an anoxic 1 M Na_2_S stock solution that was prepared as follows: Na_2_S crystals were rinsed with anoxic water, padded dry and dissolved in anoxic water by weight to a final concentration of 1 M. From this solution a ~ 50 mM sulfide stock solution was prepared in sterile anoxic water, that was acidified to the desired pH with sterile, anoxic 1 M H_2_SO_4_. These 50 mM solutions were prepared freshly on the day of use. Due to gas–liquid partitioning the final aqueous concentration in the acidified stock was approximately 30 mM. Formation of elemental sulfur occurred in these acidic sulfide stock solutions, judging from the milky appearance within seconds and settled precipitates after several hours. The subsequent chemical experiments to investigate pyrite formation and chemical sulfidogenesis from S^0^ and H_2_ were performed with either MS medium with or without YE or with DI with or without YE. In experiments where sulfide was supplemented at the start, this was done to an aqueous H_2_S concentration of 0.44 ± 0.04 mM (aq) or a total concentration of 0.72 ± 0.07 mM when total H_2_S in the liquid and gas phase are expressed over the liquid phase. Bottles were incubated statically in the dark at 40, 60, or 80 °C.

### Elemental sulfur type and commercially sourced pyrite

For the initial enrichments, colloidal chemical sulfur was used (Sigma Aldrich, St. Louis, MI), while in the sterile experiments carried out to investigate the chemical process, orthorhombic elemental sulfur (α-S^0^)^55^ was used (Sigma Aldrich, St Louis, MI). This sulfur was coarser and had a yellow color. In a later control experiment (data not shown) colloidal sulfur was found to inhibit growth of the thermoacidophilic S^0^-reducing archaeon *Acidianus ambivalens* (not shown). Although no additional compounds were listed by the manufacturer, autoclaving the colloidal sulfur powder in aqueous medium resulted in a brownish-transparent color, suggesting the presence of (an) unidentified compound(s) or the formation of sulfur intermediates. In other studies , the use of this same colloidal sulfur did not prevent enrichment of acidophilic S^0^-reducing communities at lower temperatures (pH 2–5, 30 °C)^56^, suggesting its potential inhibitory (biocidal) effects could be aggravated at higher temperatures and/or that *A. ambivalens* is more sensitive to this effect than the microbial community enriched at lower temperatures.

Several control experiments were performed using 2 mM commercially sourced pyrite (Sigma Aldrich, St. Louis, MI). This pyrite was milled in 2 mL Eppendorf tubes using a laboratory mixer mill (Retsch MM200, Retsch GmbH, Haan Germany) with a 3 mm tungsten carbide bead per Eppendorf (Qiagen GmbH, Hilden, Germany), and sieved to obtain a final particle size of < 50 μm. The powder was checked for purity by XRD before the experiment (data not shown).

### Monitoring experiments

Experiments were monitored by measuring pH and total sulfide concentration in the liquid at the time of sampling. In addition, aliquots were frozen and kept for ion chromatography and determination of dissolved iron concentrations. During sampling, bottles were kept in a water bath to maintain the corresponding experimental temperature. A pH probe designed for high temperature and low pH was used (QP150X/12 × 50/6 × 150, Prosense, Oosterhout, The Netherlands). The probe was recalibrated at room temperature in fresh buffer solutions at pH 4.0, 7.0 and 10.0 (Acros Organics, Geel, Belgium, or Hamilton, Bonaduz, Switzerland) at each sampling point and preheated to the experimental temperature before measurement. Heated buffer standard solutions were used to monitor the performance of the probe at high temperature.

At each timepoint, 1.5 mL of liquid was sampled from the bottle with a sterile 3 mL syringe and a 25G (0.5 × 25 mm) needle (Becton Dickinson and Co ltd, VV, Ireland). Part of the sample was dispensed in an Eppendorf tube, and the desired sampling volume for the methylene blue assay (100, 50, or 20 μL, depending on the expected sulfide concentration) was withdrawn with a pipet immediately to minimize loss of sulfide (see below). From the remaining sample volume, 950 μL was pipetted into a prepared Eppendorf tube containing 50 μL 99% methanol and stored at − 20 °C for determination of total dissolved iron and ion chromatography. The remaining sample volume was used to measure pH in a heating block kept at the desired temperature.

Total sulfide concentrations in the liquid phase ([H_2_S_aq_]) were measured using the methylene blue assay^57^. Briefly, an assay solution was prepared by adding 50 μL of a 5% Zn acetate solution to 9 mL demineralized water. This solution was then brought to pH > 9 with 2 M NaOH to minimize loss of sulfide. The chosen sampling volume for the methylene blue assay was pipetted into the glass reagent tube with assay solution to fix sulfide as ZnS. After sampling, 1000 μL of reagent A (2 g·L^−1^ Dimethyl-p-phenylenediamine and 200 mL·L^−1^ H_2_SO_4_) and 100 μL of reagent B (1 g·L^−1^ Fe((NH_4_)(SO_4_))^2^·12H_2_O and 0.2 mL·L^−1^ H_2_SO_4_) were added simultaneously to the sampling tubes and mixed immediately. Absorbance was measured after 10–20 min using a Spectroquant Multy colorimeter (Merck Millipore, Darmstadt, Germany) following a pre-programmed sulfide protocol (660 nm), giving a total sulfide concentration in mg·L^−1^. In subsequent data handling and plotting, values below detection limit were converted to 0.

Sulfate concentrations were determined through ion chromatography (IC) on a Dionex ICS2100, using an AS17 column (Thermo Fisher Scientific, Waltham, MA). Samples were run for 20 min at 30 °C, at 0.3 mL·min^−1^, injection volume 10 μL. Standard curves were prepared in the range of 2.5–20 mM sulfate. 30 μL of samples and standards were diluted in 970 μL 0.25 mM internal standard (KOH).

Gas samples were analyzed for H_2_ content with a Gas Chromatograph (GC) (Compact GC 4.0, Global Analyser Solutions, The Netherlands) equipped with a Carboxen 1010 pre-column and a Molsieve 5A column followed by a pulsed discharge detector (PDD). The injection oven was operated at 80 °C, the column oven at 90 °C, and the PDD at 110 °C. Helium was used as carrier gas. A H_2_ standard curve was prepared with 1% (10 000 ppm) and 4 consecutive tenfold dilutions of H_2_ gas mixtures in air. IC and GC chromatograms were analyzed with Chromeleon software (Thermo Fisher Scientific, Waltham, MA).

Ferrous iron and total dissolved iron were measured using the ferrozine assay^58^. A 1N HCl acidification step was added at the start (as described elsewhere^59^), which was later found to induce Fe^2+^ oxidation in the samples, resulting in differences between ferrous iron and total dissolved iron that were not representative of actual concentrations. This difference was not observed in the standards prepared from a 1 M FeCl_2_·4H_2_O stock solution. When the extraction step was omitted, ferrous iron and total dissolved iron in the samples were equal, and therefore total dissolved iron concentrations are taken to represent ferrous iron concentrations. This is a reasonable assumption, given the low oxidation/reduction potential ORP and sulfidic conditions in the experiments.

### Data processing and analysis and gas–liquid partitioning calculations

Data was processed in R^60^ using ggplot2^61^, ggpubr^62^ and dplyr packages in the tidyverse^63^. For data handling and plotting purposes, values below detection were converted to 0. The H_2_S concentrations in the headspace [H_2_S_gas_], and total H_2_S produced (H_2_S_total_) were calculated using the measured [H_2_S_aq_] and the temperature-corrected Henry gas–liquid partitioning coefficient (k_*H*_):

$${k}_{H}\left(T\right)= {k}_{H}^{^\circ }{e}^{\frac{d(\text{ln}({k}_{H})}{d(\frac{1}{T})}*(\frac{1}{T}-\frac{1}{298.15K})}$$

using k°_H_ = 0.1 and d(ln(k_H_))/d(1/T) = 2100 for H_2_S, and 0.00078 and 500 for H_2_, respectively as tabulated by^64^. Maximum theoretical H_2_ concentrations that could have been reached in the liquid in the case of pyrite formation through the sulfide pathway (Eq. 2) in experiments without added H_2_ were calculated using a starting amount of 0.05 mmol H_2_S added at time 0, and total liquid and gas volumes of 50 mL and 67 mL, respectively.

### Geochemical modeling

The geochemical software package PHREEQCI (Version 3.0.5–7748)^65^ was used to calculate the theoretical molar equilibrium concentrations of different sulfide species in solution at varying pH, and the saturation indices (SI) of selected iron sulfide minerals (including pyrite, pyrrhotite, mackinawite and amorphous FeS). All the calculations were conducted using the MINTEQA2 thermodynamic database^66^. This database was selected because it is the only one, among those provided with the PHREEQCI modelling package, that includes equilibrium constants for different polysulfide species (e.g. S_4_^2−^, S_5_^2−^, S_6_^2−^, …). Based on the concentrations of aqueous Fe^2+^ and H_2_S used in the experiments, the ionic activities of all dissolved constituents were calculated. The saturation indices of selected iron sulfides (pyrite, pyrrhotite, mackinawite, amorphous FeS) were calculated by using the corresponding solubility product constants included in the MINTQA2.V4 database (logK_sp_ values of − 16.81, − 4.65 and − 3.92 for pyrite, mackinawite and amorphous FeS, respectively), except for pyrrhotite (log K_sp_ = − 5.10), which is not included in this database but was taken from^67^ and manually introduced in the program. These solubility products had been previously evaluated and found to be consistent with those reported in different fractionation studies on the solubility of iron sulfides^1,60^. The assumptions for the computations (chosen to resemble the experimental conditions) are indicated in every plot.

### Electron microscopy: SEM and (S)TEM

The mineral precipitates formed in the experiments were studied by SEM–EDX analysis, and S/TEM at the SGIker Advanced Research Facilities (UPV/EHU, Bilbao, Spain). Precipitates were sampled from experiments by withdrawing 10 – 15 mL of liquid volume with sterile needles and syringes in a Coy anaerobic chamber (Coy Laboratory Products, Grass Lake, MI). Liquid was filtered over a 0.1 µm track-etch 13 mm diameter membrane filter (Whatman Nucleopore, Merck, Darmstadt DE) in a 13 mm Swinnex filter holder (Swinnex, Merck Millipore, Germany). Filters were dried on a glass Petri dish, transferred to individual 10 mL glass serum vials, sealed before removing them from the anaerobic chamber, and sent to the SGIker facilities.

Solid phases were either transferred onto double-sided adhesive carbon tape and adhered onto a SEM carbon specimen mount (Ted Pella, CA), or the sample was resuspended in ethanol and 3 µL was pipetted onto carbon tape. Prior to SEM analysis, the mount went through a plasma cleaning process of 3 min and subsequent carbon-coating. Plasma cleaning was performed in order to eliminate undesired contamination such as organics or water that could affect the image quality and to protect the microscope hardware. It was found that that plasma cleaning eliminates the S^0^, which accounted for the main part of the samples obtained from the early stage of sulfidogenesis, allowing concentrating SEM analyses on the neoformed Fe precipitates. Comparison of Fe sulfide minerals detected with and without a plasma-cleaning step suggested that this step did not affect Fe sulfide minerals.

Samples were characterized using the JSM-7000F field emission scanning electron (FEG) microscope (JEOL, Japan) working in both secondary electron (SE) and backscattered electron (BSE) modes at 20 kV beam voltage, 1 nA beam current, 10 mm working distance, vacuum < 8.35 × 10^–4^ Pa and 60 s acquisition time at every point of chemical analysis of EDX. Raw X-ray intensity values were ZAF corrected using the INCA software (Oxford Instruments, Abingdon, UK) with a set of standards for quantification.

TEM was used to further investigate the elemental composition and identify the mineral phase. For this, the solid fraction was resuspended in either ethanol or MilliQ water in an Eppendorf tube and sonicated. Afterwards, a small volume of 3–5 µL was pipetted on holey carbon-coated TEM support Cu grids (300 Mesh). Imaging, compositional point analysis and SAED analyses of neoformed sulfides were performed on Philips CM200 TEM microscope with LaB6 filament operating at 200 keV and equipped with DX-4 microanalysis system (EDAX, Pleasanton, CA). Further imaging in HAADF mode, elemental mapping and SAED was carried out on FEI Talos F200i S/TEM (ThermoFisher Scientific, Waltham, MA) operated at 200 keV and incorporating Bruker X-Flash EDX system and FEI Titan Cubed G2 60–300 kV (ThermoFisher Scientific, Waltham, MA) with a gun monochromator, a Cs-objective aberration corrector and Bruker Super-X EDX detector operated at 300 keV (Bruker, Billerica, MA).

SAED results were treated using ringGUI tool of CrysTBox software^68^. The diffraction patterns were obtained using circular averages of the image intensity as a function of the distance from the ring center in reciprocal space (g = 1/d in 1/nm). Combining direct ring measurements with the intensity profiles, the radius of these rings was converted to d-spacing and introduced in American Mineralogist Crystal Structure Database^69^. The elements and Fe:S atomic ratios detected by EDX along with the lowest possible degree of tolerance were used as constraints to determine the most probable mineral phases.

### X-ray diffraction (XRD)

For XRD analysis, samples were transferred onto a sample holder with an optional airtight seal to maintain anaerobic conditions. Analysis was tried both with and without the anaerobic seal, showing no significant influence of exposure to oxygen. Analysis of the bulk mineralogy was performed by powder X-Ray Diffraction (XRD). The XRD analysis was performed with a Bruker D8 Advance diffractometer (Bruker AXS) with Cu-Kα radiation (λ = 0.154 nm) generated at 40 kV–40 mA in the angular range 10 — 70° / 10 — 90° (2ϴ) with a step size of 0.02° and acquisition time of 1.2 s / 3 s per step, and a Lynxeye_XE_T detector. The sample was rotated during the measurement (15 rpm). The X-ray diffractogram was evaluated by the software DIFFRAC.EVA V5.2 (Bruker AXS) and plotted using OriginPro.

## Supplementary Information

Supplementary Information 1.

Supplementary Information 2.

Supplementary Figures.

Supplementary Information 4.

## Data Availability

All data generated or analysed during this study are included in this published article and its supplementary information files.

## References

[1] Rickard, D. & Luther, G. W. Chemistry of iron sulfides. *Chem. Rev.* **107**, 514–562 (2007). 17261073 10.1021/cr0503658

[2] Canfield, D. E. The evolution of the earth surface sulfur reservoir. *Am. J. Sci.* **304**, 839–861 (2004).

[3] Fike, D. A., Bradley, A. S. & Rose, C. V. Rethinking the ancient sulfur cycle. *Annu. Rev. Earth Planet. Sci.* **43**, 593–622 (2015).

[4] Wächtershäuser, G. From volcanic origins of chemoautotrophic life to Bacteria Archaea and Eukarya. *Philos. Trans. R. Soc. B Biol. Sci.* **361**, 1787–1808 (2006). 10.1098/rstb.2006.1904 PMC1664677 17008219

[5] Domingos, J. M. *et al.* Inferred pyrite growth via the particle attachment pathway in the presence of trace metals. *Geochem. Perspect. Lett.* **26**, 14–19 (2023).

[6] Gartman, A. & Luther, G. W. Comparison of pyrite (FeS_2_) synthesis mechanisms to reproduce natural FeS_2_ nanoparticles found at hydrothermal vents. *Geochim. Cosmochim. Acta* **120**, 447–458 (2013).

[7] Butler, I. B. & Rickard, D. Framboidal pyrite formation via the oxidation of iron (II) monosulfide by hydrogen sulphide. *Geochim. Cosmochim. Acta* **64**, 2665–2672 (2000).

[8] Luther, G. W. Pyrite synthesis via polysulfide compounds. *Geochim. Cosmochim. Acta* **55**, 2839–2849 (1991).

[9] Graham, U. M. & Ohmoto, H. Experimental study of formation mechanisms of hydrothermal pyrite. *Geochim. Cosmochim. Acta* **58**, 2187–2202 (1994).

[10] Wan, M., Schröder, C. & Peiffer, S. Fe(III):S(-II) concentration ratio controls the pathway and the kinetics of pyrite formation during sulfidation of ferric hydroxides. *Geochim. Cosmochim. Acta* **217**, 334–348 (2017).

[11] Rabus, R., Hansen, T. A. & Widdel, F. Dissimilatory sulfate- and sulfur-reducing prokaryotes. *The prokaryotes* **2**, 659–768 (2006).

[12] Machel, H. G. Bacterial and thermochemical sulfate reduction in diagenetic settings - old and new insights. *Sediment. Geol.* **140**, 143–175 (2001).

[13] Truche, L., Berger, G., Destrigneville, C., Guillaume, D. & Giffaut, E. Kinetics of pyrite to pyrrhotite reduction by hydrogen in calcite buffered solutions between 90 and 180°C: Implications for nuclear waste disposal. *Geochim. Cosmochim. Acta* **74**, 2894–2914 (2010).

[14] Payne, D., Spietz, R. L. & Boyd, E. S. Reductive dissolution of pyrite by methanogenic archaea. *ISME J.* **15**, 3498–3507 (2021). 34112969 10.1038/s41396-021-01028-3 PMC8630215

[15] Spietz, R. L. *et al.* Investigating abiotic and biotic mechanisms of pyrite reduction. *Front. Microbiol.* **13**, 878387 (2022). 35615515 10.3389/fmicb.2022.878387 PMC9124975

[16] Amenabar, M. J., Shock, E. L., Roden, E. E., Peters, J. W. & Boyd, E. S. Microbial substrate preference dictated by energy demand rather than supply. *Nat. Geosci.* **10**, 577–581 (2017). 30944580 10.1038/ngeo2978 PMC6443248

[17] Belkin, S., Wirsen, C. O. & Jannasch, H. W. Biological and abiological sulfur reduction at high temperatures. *Appl. Environ. Microbiol.* **49**, 1057–1061 (1985). 16346781 10.1128/aem.49.5.1057-1061.1985 PMC238504

[18] Finster, K. Microbiological disproportionation of inorganic sulfur compounds. *J. Sulfur Chem.* **29**, 281–292 (2008).

[19] Faber, M. S., Lukowski, M. A., Ding, Q., Kaiser, N. S. & Jin, S. Earth-abundant metal pyrites (FeS_2_, CoS_2_, NiS_2_, and their alloys) for highly efficient hydrogen evolution and polysulfide reduction electrocatalysis. *J. Ofc Phys. Chem. C* **118**, 21347–21356 (2014). 10.1021/jp506288w PMC4167051 25247028

[20] Faber, M. S. & Jin, S. Earth-abundant inorganic electrocatalysts and their nanostructures for energy conversion applications. *Energy Environ. Sci.* **7**, 3519–3542 (2014).

[21] Truche, L. *et al.* Sulphide mineral reactions in clay-rich rock induced by high hydrogen pressure. Application to disturbed or natural settings up to 250°C and 30bar. *Chem. Geol.* **351**, 217–228 (2013).

[22] Hassannayebi, N., Azizmohammadi, S., De Lucia, M. & Ott, H. Underground hydrogen storage: Application of geochemical modelling in a case study in the Molasse Basin, Upper Austria. *Environ. Earth Sci.* **78**, 1–14 (2019).

[23] Ma, B. *et al.* Pyrite nanoparticles: An Earth-abundant mineral catalyst for activation of molecular hydrogen and hydrogenation of nitroaromatics. *RSC Adv.* **6**, 55220–55224 (2016).

[24] Adhikari, M., Singh, A., Echeverria, E., McIlroy, D. N. & Vasquez, Y. Iron pyrite nanocrystals: A potential catalyst for selective transfer hydrogenation of functionalized nitroarenes. *ACS Omega* **5**, 14104–14110 (2020). 32566877 10.1021/acsomega.0c01637 PMC7301598

[25] Nakamura, R. *et al.* Electrical current generation across a black smoker chimney. *Angew. Chem. - Int. Ed.* **49**, 7692–7694 (2010). 10.1002/anie.201003311 20839203

[26] Yamamoto, M. *et al.* Spontaneous and widespread electricity generation in natural deep-sea hydrothermal fields. *Angew. Chem.* **129**, 5819–5822 (2017). 10.1002/anie.201701768 28378459

[27] Yamamoto, M., Nakamura, R. & Takai, K. Deep-sea hydrothermal fields as natural power plants. *ChemElectroChem* **5**, 2162–2166 (2018).

[28] Nielsen, L. P., Risgaard-Petersen, N., Fossing, H., Christensen, P. B. & Sayama, M. Electric currents couple spatially separated biogeochemical processes in marine sediment. *Nature* **463**, 1071–1074 (2010). 20182510 10.1038/nature08790

[29] Morse, J. W. & Wang, Q. Pyrite formation under conditions approximating those in anoxic sediments: II. Influence of precursor iron minerals and organic matter. *Mar. Chem.* **57**, 187–193 (1997).

[30] ThomasArrigo, L. K., Bouchet, S., Kaegi, R. & Kretzschmar, R. Organic matter influences transformation products of ferrihydrite exposed to sulfide. *Environ. Sci. Nano* **7**, 3405–3418 (2020).

[31] Wang, W., Song, Y., Wang, X., Yang, Y. & Liu, X. Alpha-oxo acids assisted transformation of FeS to Fe_3_S_4_ at low temperature: implications for abiotic, biotic, and prebiotic mineralization. *Astrobiology* **15**, 1043–1051 (2015). 26625153 10.1089/ast.2015.1373

[32] Renock, D. *et al.* Chemical and structural characterization of As immobilization by nanoparticles of mackinawite (FeSm). *Chem. Geol.* **268**, 116–125 (2009).

[33] Boyd, E. S. & Druschel, G. K. Involvement of intermediate sulfur species in biological reduction of elemental sulfur under acidic, hydrothermal conditions. *Appl. Environ. Microbiol.* **79**, 2061–2068 (2013). 23335768 10.1128/AEM.03160-12 PMC3592231

[34] Reimer, L. & Kohl, H. *Transmission Electron Microscopy: Physics of Image Formation* (Springer, 2008).

[35] Rickard, D. *et al.* The composition of nanoparticulate mackinawite, tetragonal iron(II) monosulfide. *Chem. Geol.* **235**, 286–298 (2006).

[36] Amend, J. P. & Shock, E. L. Energetics of overall metabolic reactions of thermophilic and hyperthermophilic *Archaea* and *Bacteria*. *FEMS Microbiol. Rev.* **25**, 175–243 (2001). 11250035 10.1111/j.1574-6976.2001.tb00576.x

[37] Kamyshny, A., Gun, J., Rizkov, D., Voitsekovski, T. & Lev, O. Equilibrium distribution of polysulfide ions in aqueous solutions at different temperatures by rapid single phase derivatization. *Environ. Sci. Technol.* **41**, 2395–2400 (2007). 17438792 10.1021/es062637+

[38] Kamyshny, A., Goifman, A., Gun, J., Rizkov, D. & Lev, O. Equilibrium distribution of polysulfide ions in aqueous solutions at 25°C: A new approach for the study of polysulfides’ equilibria. *Environ. Sci. Technol.* **38**, 6633–6644 (2004). 15669322 10.1021/es049514e

[39] Rickard, D. Kinetics of pyrite formation by the H_2_S oxidation of iron (II) monosulfide in aqueous solutions between 25 and 125°C: The rate equation. *Geochim. Cosmochim. Acta* **61**, 115–134 (1997).

[40] Kafantaris, F.-C.A. & Druschel, G. K. Kinetics of the nucleophilic dissolution of hydrophobic and hydrophilic elemental sulfur sols by sulfide. *Geochim. Cosmochim. Acta* **269**, 554–565 (2020).

[41] Berner, R. A. The synthesis of framboidal pyrite. *Econ. Geol.* **64**, 383–384 (1969).

[42] Ohfuji, H. & Rickard, D. Experimental syntheses of framboids - A review. *Earth-Sci. Rev.* **71**, 147–170 (2005).

[43] Wang, Q. & Morse, J. W. Pyrite formation under conditions approximating those in anoxic sediments: I Pathway and morphology. *Mar. Chem.* **52**, 99–121 (1996).

[44] Sánchez-España, J. Crystallization in acidic media: From nanoparticles to macrocrystals. *Crystal. Under Extreme Condit.* **13**, 15–34 (2017).

[45] Duverger, A., Bernard, S., Viennet, J., Miot, J. & Busigny, V. Formation of pyrite spherules from mixtures of biogenic FeS and organic compounds during experimental diagenesis. *Geochem. Geophys. Geosystems* **22**, 1–16 (2021).

[46] Matamoros-Veloza, A. *et al.* A highly reactive precursor in the iron sulfide system. *Nat. Commun.* **9**, (2018). 10.1038/s41467-018-05493-x PMC6081449 30087338

[47] Rickard, D. Metal sulfide complexes and clusters. *Rev. Mineral. Geochem.* **61**, 421–504 (2006).

[48] Vaughan, D. J. & Coker, V. S. Biogeochemical redox processes of sulfide minerals. in *Redox-reactive Minerals: Properties, Reactions and Applications in Clean Technologies* (eds. Ahmed, I. A. M. & Hudson-Edwards, K. A.) vol. 17 0 (European Mineralogical Union, 2017).

[49] Yücel, M., Gartman, A., Chan, C. S. & Luther, G. W. Hydrothermal vents as a kinetically stable source of iron-sulphide-bearing nanoparticles to the ocean. *Nat. Geosci.* **4**, 367–371 (2011).

[50] Gartman, A., Findlay, A. J. & Luther, G. W. Nanoparticulate pyrite and other nanoparticles are a widespread component of hydrothermal vent black smoker emissions. *Chem. Geol.* **366**, 32–41 (2014).

[51] Truche, L., & Bazarkina, E. F. Natural hydrogen the fuel of the 21st century. *E3S Web Conf.* **98**, 03006 (2019).

[52] Zgonnik, V. The occurrence and geoscience of natural hydrogen: A comprehensive review. *Earth-Sci. Rev.* **203**, 103140 (2020).

[53] Stams, A. J. M., Van Dijk, J. B., Dijkema, C. & Plugge, C. M. Growth of syntrophic propionate-oxidizing bacteria with fumarate in the absence of methanogenic bacteria. *Appl. Environ. Microbiol.* **59**, 1114–1119 (1993). 16348912 10.1128/aem.59.4.1114-1119.1993 PMC202247

[54] McCleskey, B., Ball, J. W., Nordstrom, D. K., Holloway, J. M. & Taylor, H. E. *Water-Chemistry Data for Selected Hot Springs, Geysers, and Streams in Yellowstone National Park, Wyoming*. (2005).

[55] Steudel, R. The chemical sulfur cycle. in *Environmental Technologies to Treat Sulphur Pollution: Principles and Engineering* 11–53 (IWA Publishing, 2020). 10.2166/9781789060966_0011.

[56] Florentino, A. P., Weijma, J., Stams, A. J. M. & Sánchez-Andrea, I. Sulfur reduction in acid rock drainage environments. *Environ. Sci. Technol.* **49**, 11746–11755 (2015). 26356416 10.1021/acs.est.5b03346

[57] Cline, J. Spectrophotometric determination of hydrogen sulfide in natural waters. *Limnol. Oceanogr.* **14**, 454–458 (1969).

[58] Stookey, L. L. Ferrozine-a new spectrophotometric reagent for iron. *Anal. Chem.* **42**, 779–781 (1970).

[59] Janssen, P. H., Schuhmann, A., Bak, F. & Liesack, W. Disproportionation of inorganic sulfur compounds by the sulfate-reducing bacterium *Desulfocapsa thiozymogenes* gen, nov., sp. nov. *Arch. Microbiol.* **166**, 184–192 (1996).

[60] R Core Team. R: A language and environment for statistical computing. (R Foundation for Statistical Computing, Vienna, Austria, 2021). https://www.R-project.org.

[61] Wickham, H. *Elegant Graphics for Data Analysis: Ggplot2*. (Springer-Verlag, New York, 2016). ISBN 978-3-319-24277-4. https://ggplot2.tidyverse.org.

[62] Kassambara, Alboukadel. ggpubr:“ggplot2” based publication ready plots (2020). https://rpkgs.datanovia.com/ggpubr

[63] Wickham, H. *et al.* Welcome to the Tidyverse. *J. Open Source Softw.* **4**, 1686 (2019).

[64] Sander, R. Compilation of Henry’s law constants (version 4.0) for water as solvent. *Atmospheric Chem. Phys.* **15**, 4399–4981 (2015).

[65] Parkhurst, D. L. & Appelo, C. A. J. User’s guide to PHREEQC (Version 2): a computer program for speciation, batch-reaction, one-dimensional transport, and inverse geochemical calculations. *Water-Resour. Investig. Rep.*10.3133/wri994259 (1999).

[66] Allison, J. D., Brown, D. S. & Novo-Gradac, K. J. MINTEQA2/PRODEFA2, a geochemical assessment model for environmental systems: version 3.0 user’s manual. *Environ. Res. Lab. Off. Res. Dev. US Environ. Prot. Agency* (1991).

[67] Davison, W. The solubility of iron sulphides in synthetic and natural waters at ambient temperature. *Aquat. Sci.* **53**, 309–329 (1991).

[68] Klinger, M. & Jäger, A. *Crystallographic Tool Box* ( *CrysTBox* ): automated tools for transmission electron microscopists and crystallographers. *J. Appl. Crystallogr.* **48**, 2012–2018 (2015). 26664349 10.1107/S1600576715017252 PMC4665667

[69] Downs, R. T. & Hall-Wallace, M. The American mineralogist crystal structure database. *Am. Mineral.* **88**, 247–250 (2003).

