# Supplementary figures and images for: Micrometric pyrite catalyzes abiotic sulfidogenesis from elemental sulfur and hydrogen
Source: Sci Rep. 2024 Jul 31;14:17702. doi: 10.1038/s41598-024-66006-z (PMC11291890; doi:10.1038/s41598-024-66006-z)

48

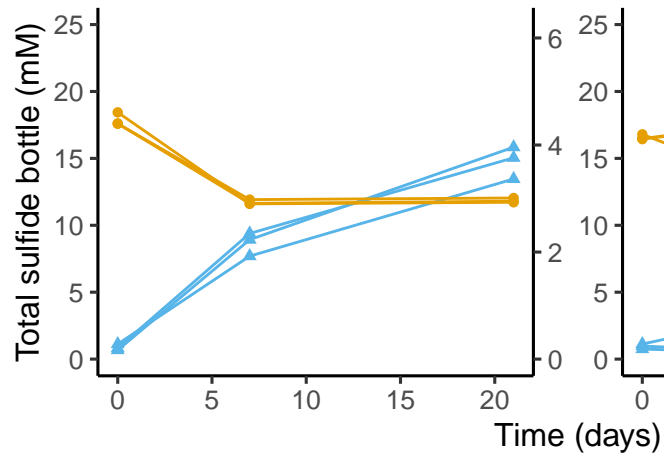

49

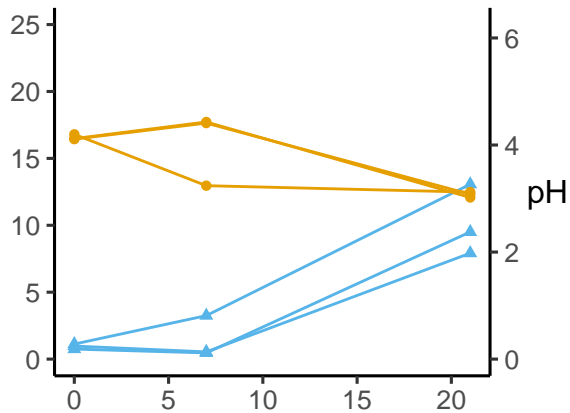

parameter

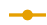

pH

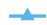

Stotal\_mM\_liq

Supplement: Supplementary file 2 — Supplementary Information 2. [file 41598_2024_66006_MOESM2_ESM.zip › vandergraaf_et_al2023_pyrite/figures/paper/SIFig5_salinity.pdf]

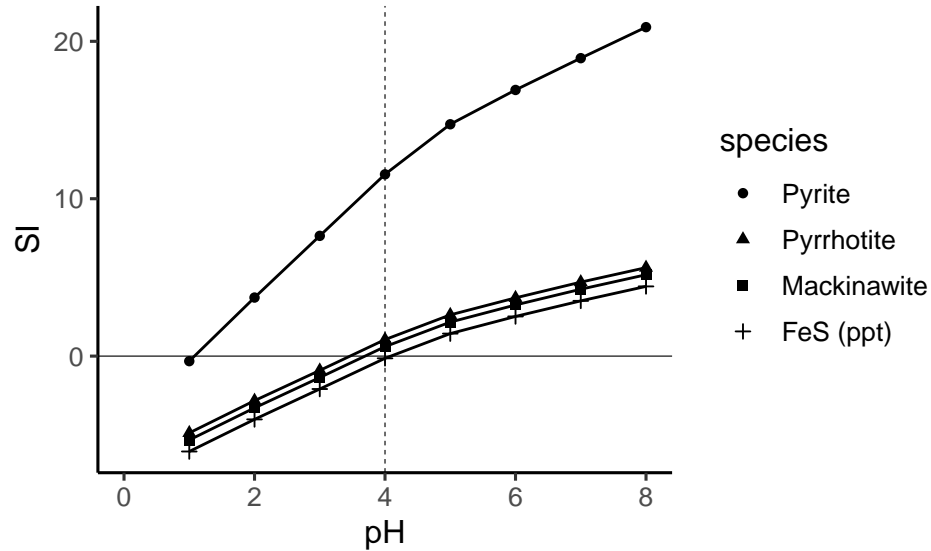

Supplement: Supplementary file 2 — Supplementary Information 2. [file 41598_2024_66006_MOESM2_ESM.zip › vandergraaf_et_al2023_pyrite/figures/paper/fig3b_phr_fes_si_pH_higherH2S.pdf]

# Total sulfide (aq)

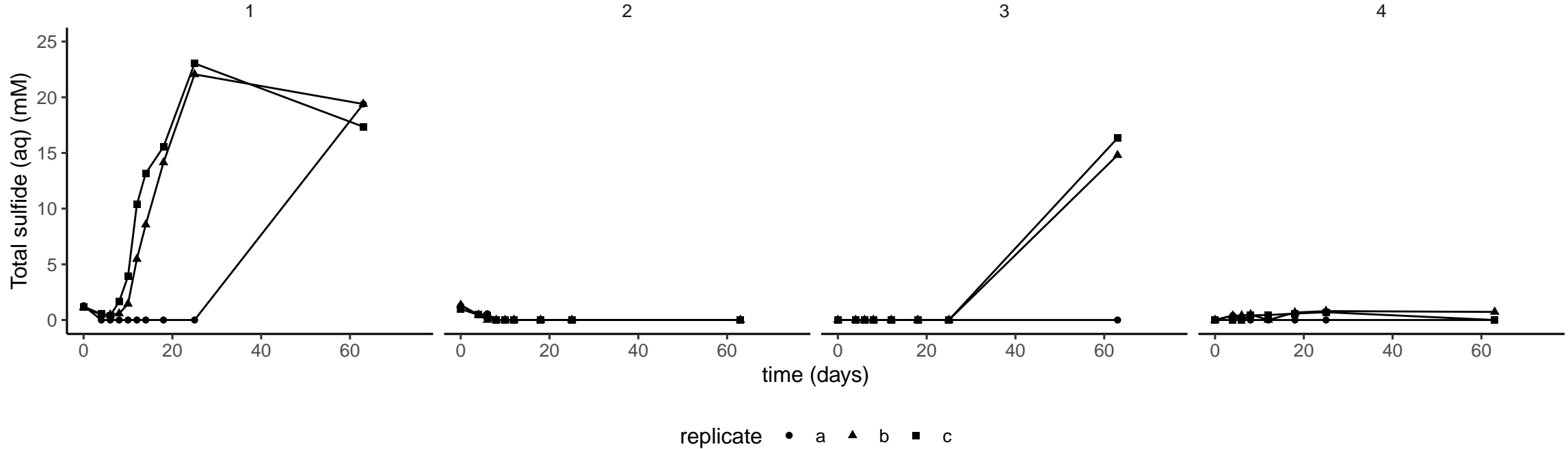

Supplement: Supplementary file 2 — Supplementary Information 2. [file 41598_2024_66006_MOESM2_ESM.zip › vandergraaf_et_al2023_pyrite/figures/paper/SIFig1_ctasre1_H2S.pdf]

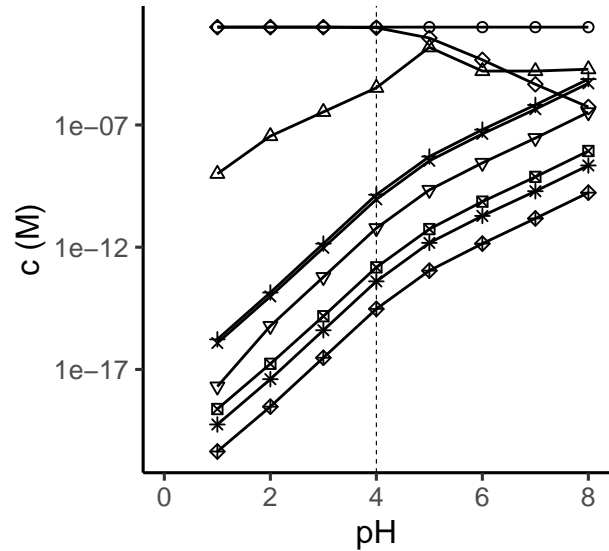

Supplement: Supplementary file 2 — Supplementary Information 2. [file 41598_2024_66006_MOESM2_ESM.zip › vandergraaf_et_al2023_pyrite/figures/paper/fig3a_phr_polysulfides_pH.pdf]

48

49

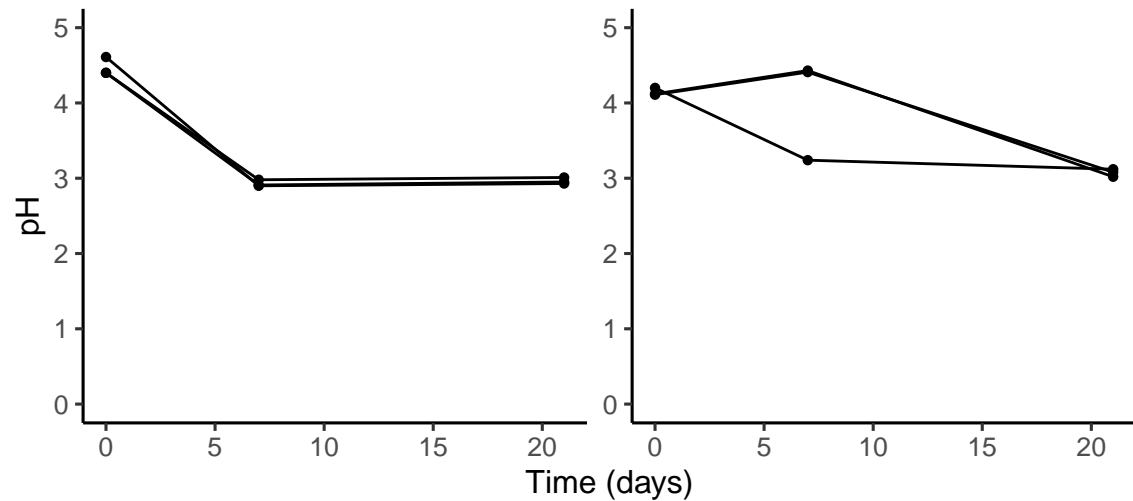

parameter

• pH

Supplement: Supplementary file 2 — Supplementary Information 2. [file 41598_2024_66006_MOESM2_ESM.zip › vandergraaf_et_al2023_pyrite/figures/paper/SI_Fig5B_salinity_pH.pdf]

48

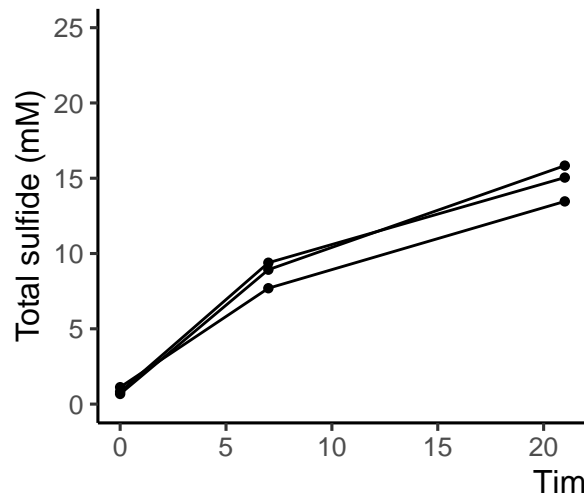

49

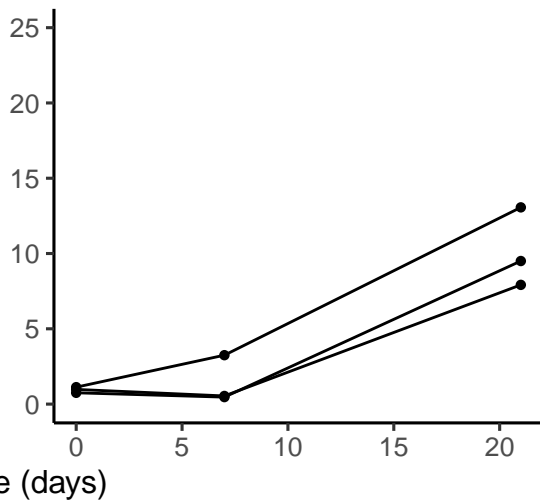

parameter

• Stotal\_mM\_liq

Supplement: Supplementary file 2 — Supplementary Information 2. [file 41598_2024_66006_MOESM2_ESM.zip › vandergraaf_et_al2023_pyrite/figures/paper/SI_Fig5A_salinity_H2S.pdf]

Total sulfide (aq) (mM)

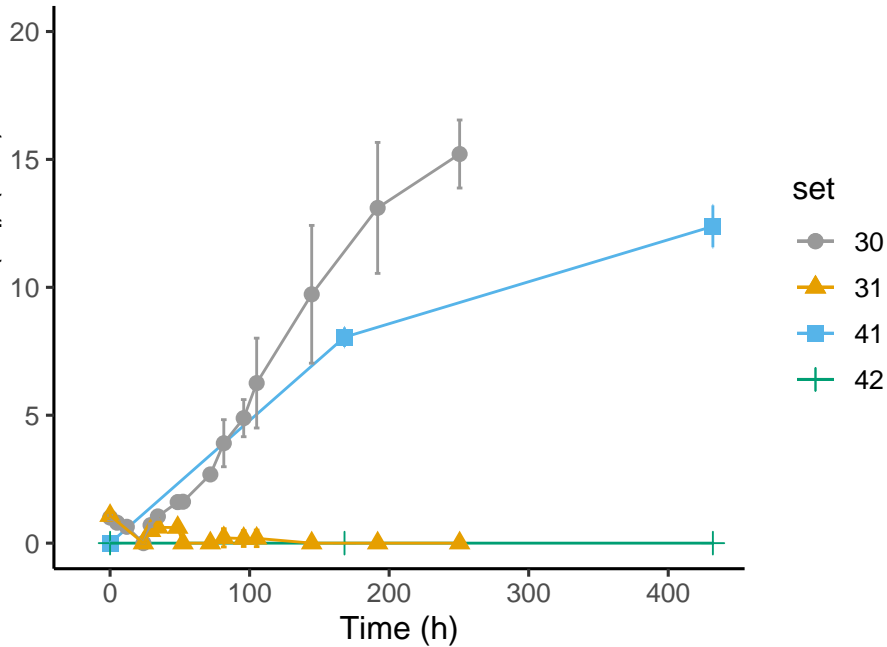

Supplement: Supplementary file 2 — Supplementary Information 2. [file 41598_2024_66006_MOESM2_ESM.zip › vandergraaf_et_al2023_pyrite/figures/paper/fig1a_combined_H2S.pdf]

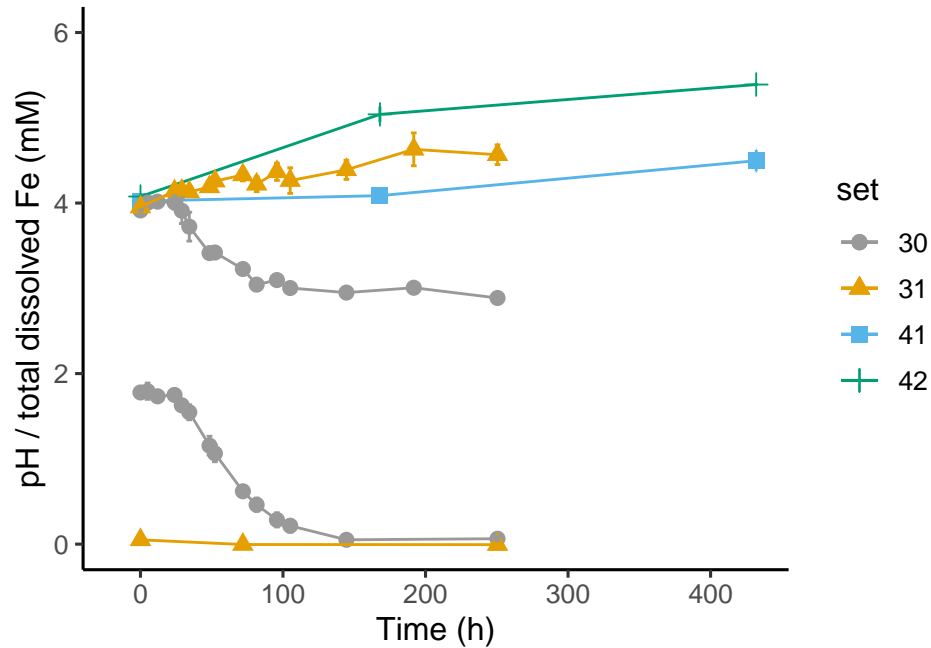

Supplement: Supplementary file 2 — Supplementary Information 2. [file 41598_2024_66006_MOESM2_ESM.zip › vandergraaf_et_al2023_pyrite/figures/paper/fig1b_combined_pH-Fe.pdf]

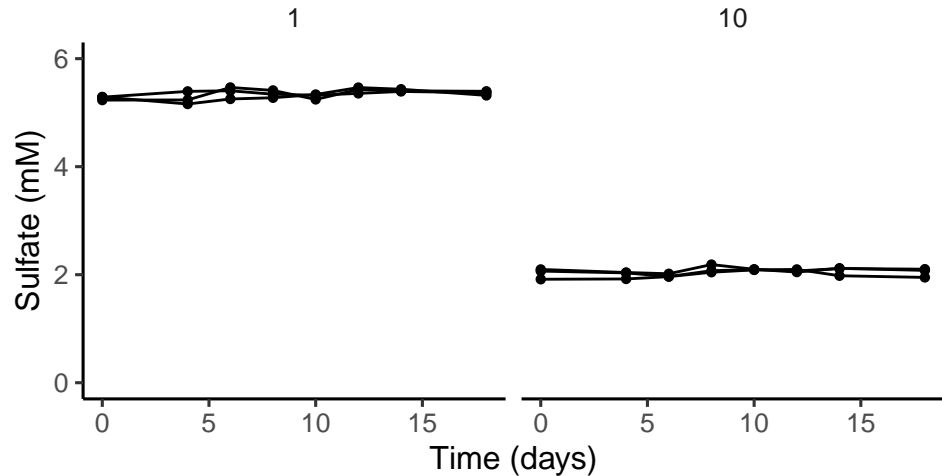

compound

• SO<sub>4</sub>

Supplement: Supplementary file 2 — Supplementary Information 2. [file 41598_2024_66006_MOESM2_ESM.zip › vandergraaf_et_al2023_pyrite/figures/paper/SI_Fig3.pdf]

11

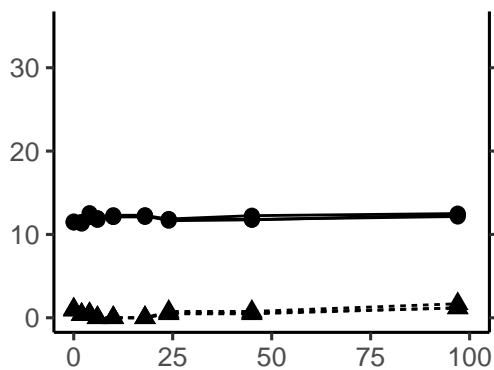

12

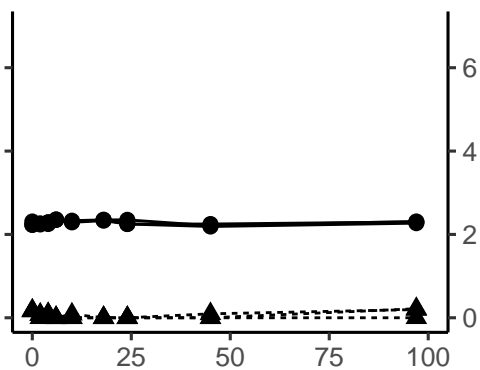

13

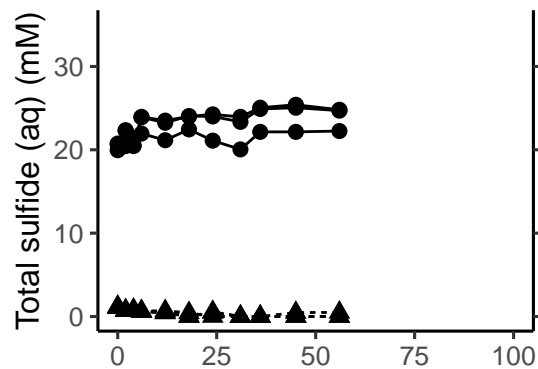

14

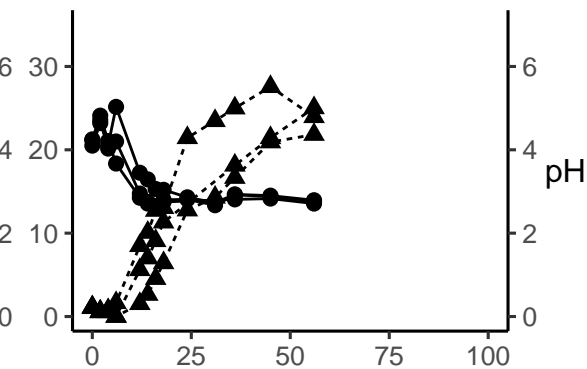

15

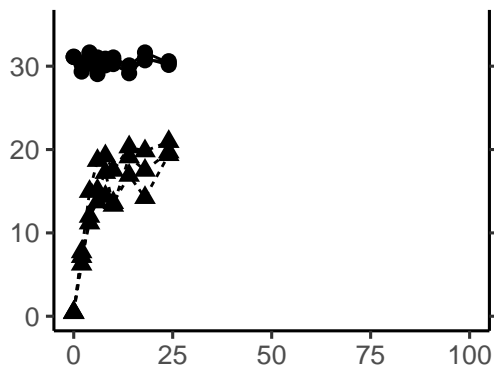

16

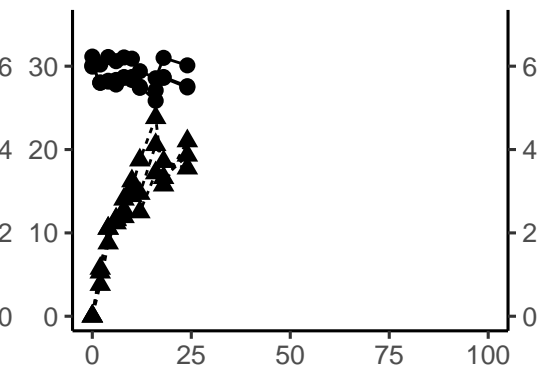

Time (days)

parameter

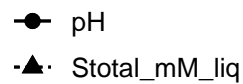

Supplement: Supplementary file 2 — Supplementary Information 2. [file 41598_2024_66006_MOESM2_ESM.zip › vandergraaf_et_al2023_pyrite/figures/paper/SI_Fig2.pdf]

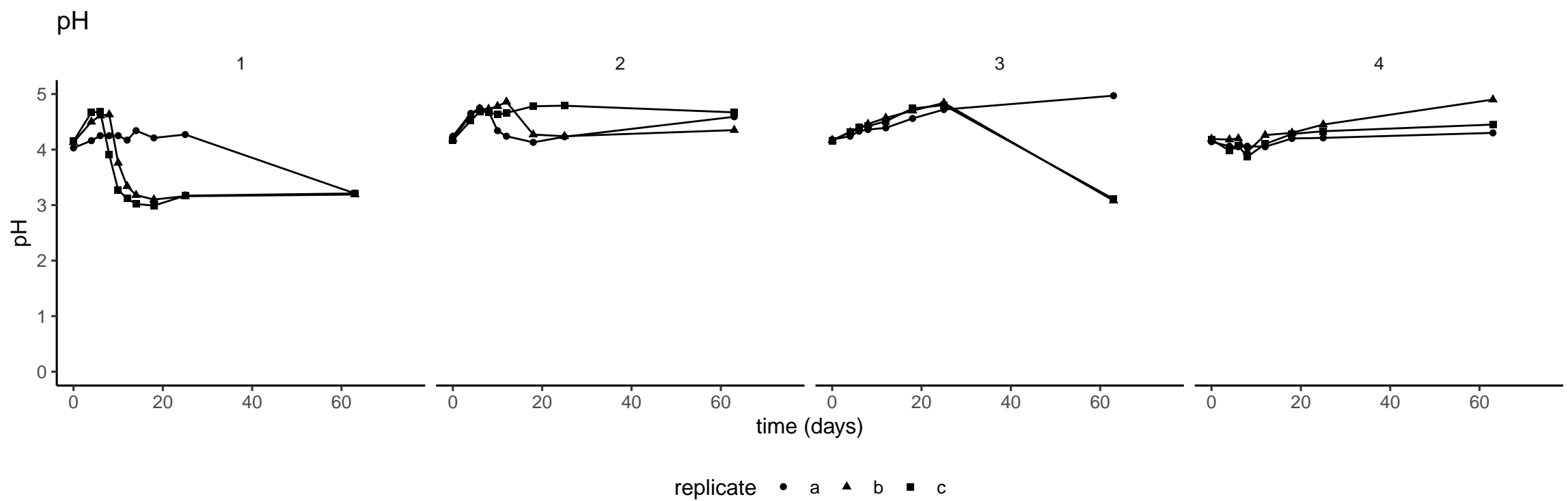

Supplement: Supplementary file 2 — Supplementary Information 2. [file 41598_2024_66006_MOESM2_ESM.zip › vandergraaf_et_al2023_pyrite/figures/paper/SIFig1_ctasre1_pH.pdf]
