# Supplementary material for: Micrometric pyrite catalyzes abiotic sulfidogenesis from elemental sulfur and hydrogen
Source: Sci Rep. 2024 Jul 31;14:17702. doi: 10.1038/s41598-024-66006-z (PMC11291890; doi:10.1038/s41598-024-66006-z)
Supplement: Supplementary file 3 — Supplementary Figures. [file 41598_2024_66006_MOESM3_ESM.pdf]

Supplementary Figures

Micrometric pyrite catalyzes abiotic sulfidogenesis from elemental sulfur and hydrogen

*Charlotte M. van der Graaf [1,2 *], Javier Sánchez-España[3], Andrey M. Ilin[4], Iñaki Yusta [4], Alfons J. M. Stams [1,54], Irene Sánchez-Andrea [1,6]*

[1] Laboratory of Microbiology, Wageningen University, Stippeneng 4, 6708 WE Wageningen, The Netherlands; [2] Delft University of Technology, Faculty of Civil Engineering and Geoscience, Department of Geoscience and Engineering, Stevinweg 1 - 2628CN Delft; [3] Planetary Geology Research Group, Department of Planetology and Habitability, Centro de Astrobiología (CAB, CSIC-INTA), 28850 Torrejón de Ardoz, Madrid, Spain; [4] Department of Geology, University of the Basque Country (UPV/EHU), Apdo. 644, 48080, Bilbao, Spain; [5] Centre of Biological Engineering, University of Minho, Campus de Gualtar, 4710-057 Braga, Portugal; [6] *IE university, Department of Environmental Sciences for Sustainability, C. Cardenal Zúñiga, 12, 40003 Segovia, Spain*

[*] *Corresponding author: c.m.vandergraaf@tudelft.nl*

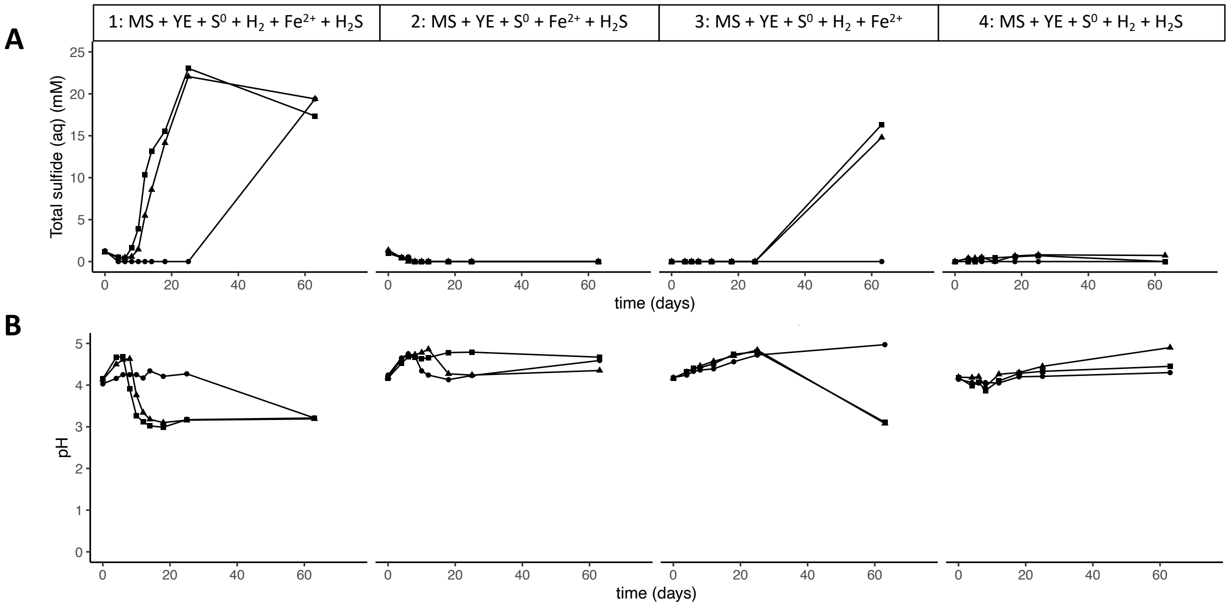

Supplementary Figure s1 | Sulfide production in incubations resembling a microbial batch growth curve, and negative controls. A. total sulfide concentrations expressed over the remaining liquid volume, and **B.** pH in triplicate incubations with minimal salts (MS) medium + yeast extract (YE), S^0 and (1) H_2, Fe^{2+}, and H_2S supplemented at the start of the incubations; (2) Fe^{2+} and H_2S supplementation, but no H_2; (3) H_2 and Fe^{2+}, but no H_2S supplemented at the start of the incubations*; (4) H_2 and H_2S but no Fe^{2+} supplemented at the start of the incubations.

* We hypothesize that the production of H_2S measured after 68 days in incubations with no added H_2S is due to the production of very small amounts of polysulfides at the surface of the elemental sulfur particles during prolonged incubation at high temperatures. These polysulfides would then decompose to sulfide and nanosulfur due to the instability of polysulfides at low pH [1,2]. This could provide a route for initial formation of low amounts of H_2S in these incubations, which then enable formation of pyrite, starting the catalysis of sulfidogenesis from H_2 and S^0 .

This hypothesis is supported by an observation we made in a different experiment (data not shown), where in incubations with only S^0 and H_2, but no Fe^{2+}, that were left for over 60 days, H_2S concentrations were below detection, but sulfidogenesis started within 12 hours after addition of Fe^{2+} to these 'old' incubations (qualitative observation). This was done as a quick test, in the period where we were still thinking that our 'enrichment' incubations were microbial, and possible Fe^{2+} limitation could be a factor. No rigorous time series were made for H_2S concentrations in this incubation, but in hindsight we interpret these results as supporting the formation of low amounts of H_2S after prolonged incubation.

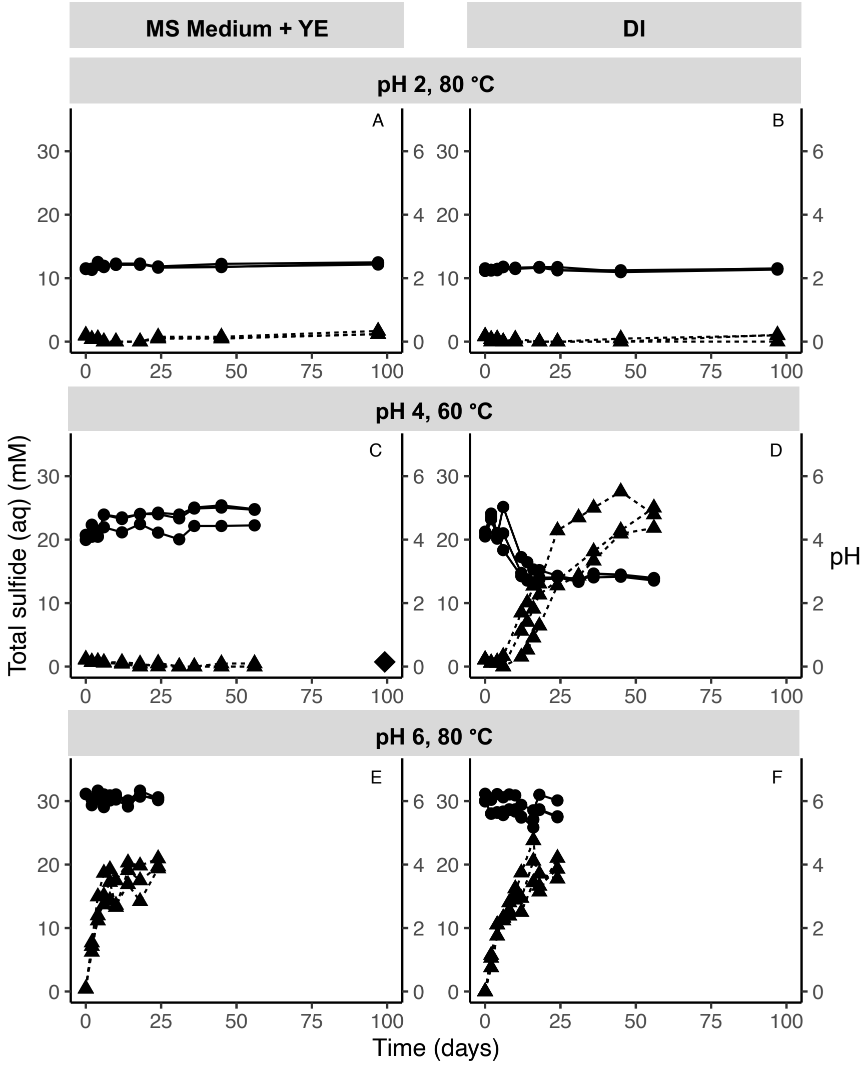

Supplementary Figure s2 | Sulfide production (triangles, left axes) and pH (circles, right axes) in incubations with MS medium + YE (left column) or deionized water (DI) (right column) and S^0, H_2, Fe^{2+} and H_2S incubated at different combinations of starting pH and temperature. A, B. starting pH 2, 80 °C; **C, D.** starting pH 4, 60 °C. Checker symbol indicates that visual inspection on day 285 indicated all S^0 was removed, and a fine black precipitate remained; **E, F.** starting pH 6, 80 °C.

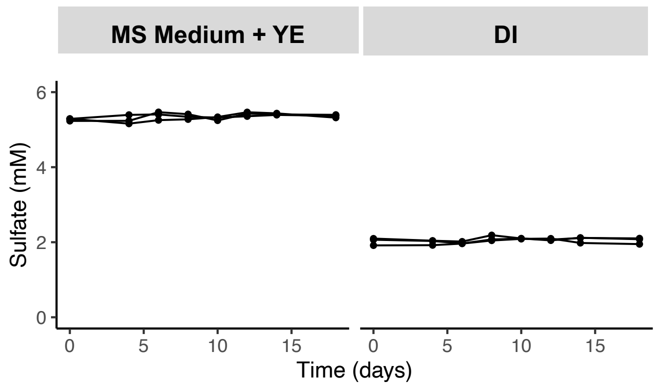

Supplementary Figure s3 | Sulfate concentrations in sulfidogenic incubations*. A. MS medium + YE **+ S^0 + Fe^{2+} + H_2S + H_2** (compare figure 1A) and **B.** acidified demineralized water (DI) **+ S^0 + Fe^{2+} + H_2S + H_2** during the first 18 days of incubation. Individual replicates are shown.

* Sulfate concentrations in DI originate from the H_2SO_4 used to set the pH. Sulfate concentrations in the MS medium were 3.3 mM before pH correction, the remainder results from adjustment of pH with H_2SO_4.

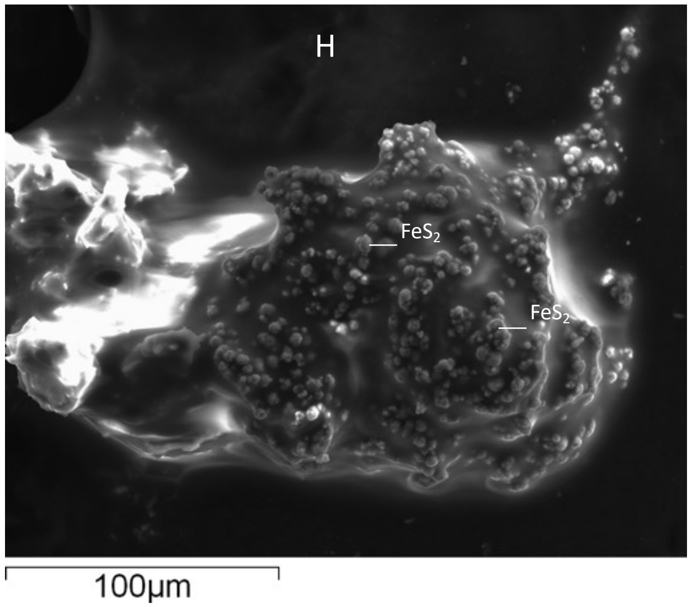

Supplementary Figure s4 | iron sulfide particles with a 1:2 ratio, indicating pyrite, detected on sulfur grains in MS medium + YE + S^0 + Fe^{2+} + H_2S without H_2, harvested on day 8.

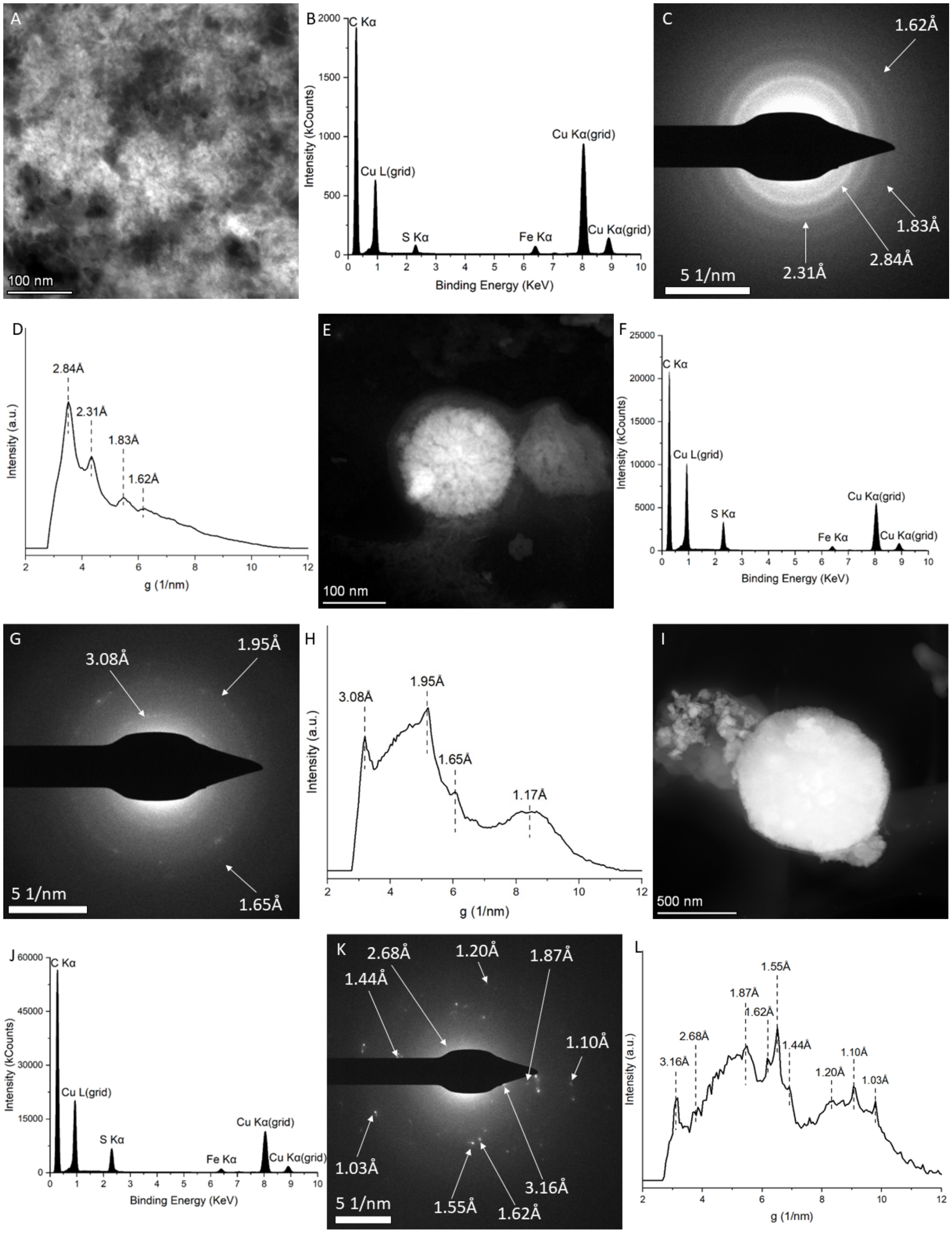

Supplementary Figure s5 | STEM micrographs of iron sulfides extracted from different incubations with MS medium + S^0 + Fe^{2+} + H_2 + H_2S and YE (A-D) or no YE (E-L). (A-D) Acicular iron monosulfide cluster harvested after 8 days. (E-H) Spherical iron disulfide aggregate harvested after 4 days. (I-L) Spherical porous iron disulfide aggregate harvested after 1 day. For each ROI, HAADF, EDS, SAED and corresponding color profile are provided.

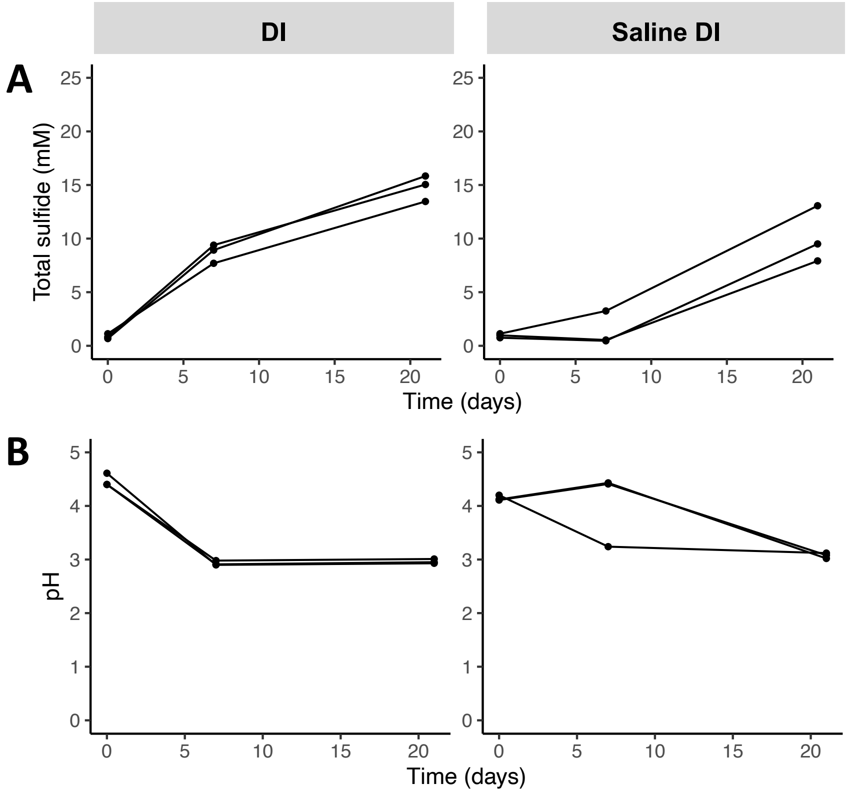

Supplementary Figure s6 | Effect of increased salinity on sulfide production in incubations with deionized water (DI) + S^0 + Fe^{2+} + H_2 + H_2S without (left column) or with 3 g/L Na_2SO_4 and 21 g/L NaCl (right). A. total H_2S expressed in mM over the remaining liquid and **B.** pH.

References

1. Kamyshny, A., Goifman, A., Gun, J., Rizkov, D. & Lev, O. Equilibrium distribution of polysulfide ions in aqueous solutions at 25 °C: a new approach for the study of polysulfides' equilibria. *Environ. Sci. Technol.* **38**, 6633–6644 (2004).
2. Kamyshny, A., Gun, J., Rizkov, D., Voitsekovski, T. & Lev, O. Equilibrium Distribution of Polysulfide Ions in Aqueous Solutions at Different Temperatures by Rapid Single Phase Derivatization. *Environ. Sci. Technol.* **41**, 2395–2400 (2007).
