# Supplementary material for: Micrometric pyrite catalyzes abiotic sulfidogenesis from elemental sulfur and hydrogen
Source: Sci Rep. 2024 Jul 31;14:17702. doi: 10.1038/s41598-024-66006-z (PMC11291890; doi:10.1038/s41598-024-66006-z)

Supplementary File 1

Micrometric pyrite catalyzes abiotic sulfidogenesis from elemental sulfur and hydrogen

*Charlotte M. van der Graaf [1,2 *], Javier Sánchez-España[3], Andrey M. Ilin[4], Iñaki Yusta [4], Alfons J. M. Stams [1,54], Irene Sánchez-Andrea [1,6]*

[1] Laboratory of Microbiology, Wageningen University, Stippeneng 4, 6708 WE Wageningen, The Netherlands; [2] Delft University of Technology, Faculty of Civil Engineering and Geoscience, Department of Geoscience and Engineering, Stevinweg 1 - 2628CN Delft; [3] Planetary Geology Research Group, Department of Planetology and Habitability, Centro de Astrobiología (CAB, CSIC-INTA), 28850 Torrejón de Ardoz, Madrid, Spain; [4] Department of Geology, University of the Basque Country (UPV/EHU), Apdo. 644, 48080, Bilbao, Spain; [5] Centre of Biological Engineering, University of Minho, Campus de Gualtar, 4710-057 Braga, Portugal; [6] *IE university, Department of Environmental Sciences for Sustainability, C. Cardenal Zúñiga, 12, 40003 Segovia, Spain*

[*] *Corresponding author: c.m.vandergraaf@tudelft.nl*

LM6109 st03   FIGURE 1A

Processing option : All elements analysed (Normalised)

Spectrum	In stats.	O	Na	Si	S	K	Fe	**Fe:S**
Spectrum 1	Yes	17.86		2.81	55.67		23.66	**1 : 2.35**
Spectrum 2	Yes	31.00		6.25	62.75			
Spectrum 3	Yes	9.14		16.00	74.86			
Spectrum 4	Yes	30.32	0.99	4.10	45.53	0.26	18.80	**1 : 2.42**

All results in atomic%

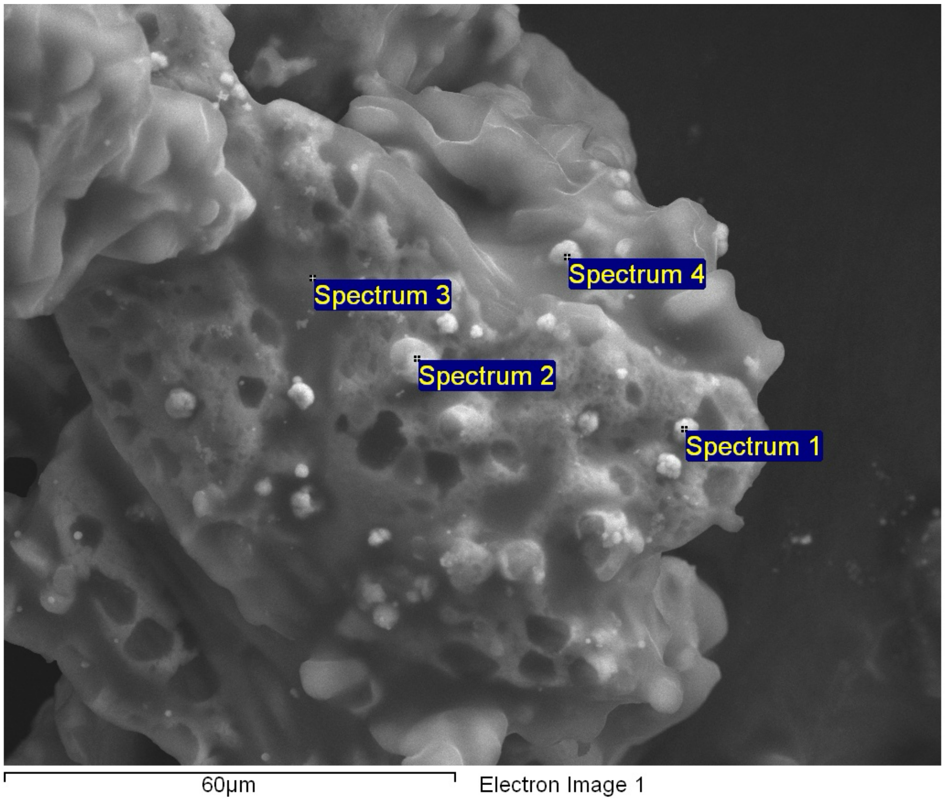

Electron Image 1

LM61c03 st02 FIGURE 1B

Processing option : All elements analysed (Normalised)

Spectrum	In stats.	O	Na	Si	S	K	Ca	Fe	**Fe:S**
Spectrum 1	Yes	19.99	0.88	2.60	50.66			25.87	
Spectrum 2	Yes	48.55	10.08	4.47	24.35	0.45	1.42	10.68	**1 : 2.28**

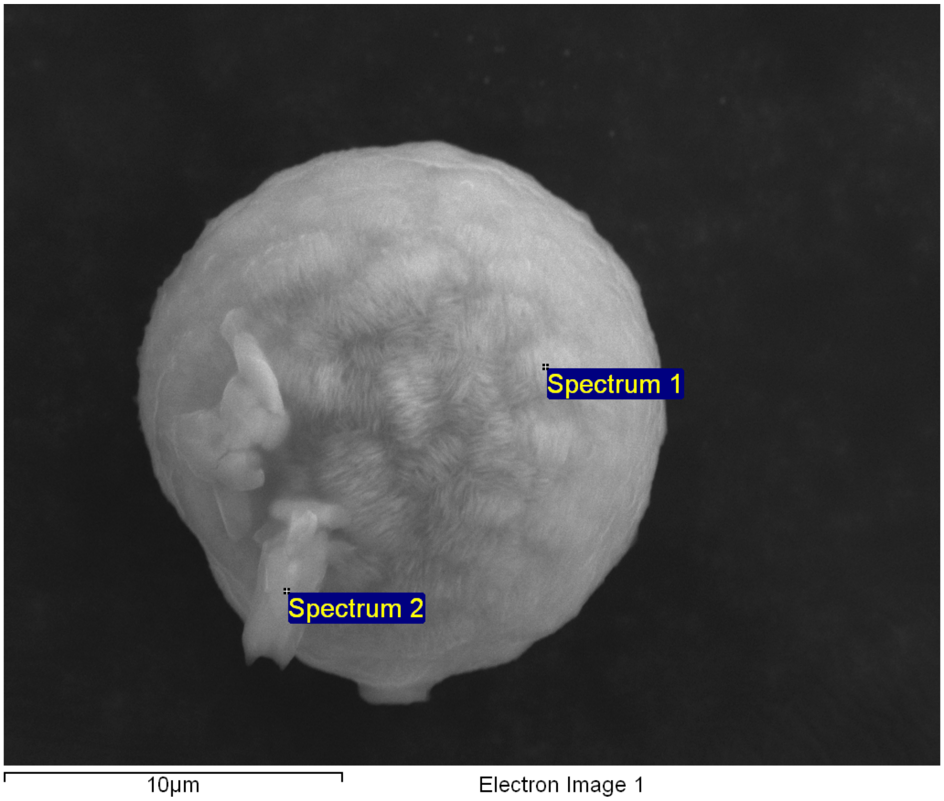

LM61c03 st01   FIGURE 1B-insert

Spectrum	In stats.	O	Na	Si	S	Fe	**Fe:S**
Spectrum 1	Yes	20.78	0.75	2.15	50.73	25.59	**1 : 1.98**
Spectrum 2	Yes	16.60		1.49	56.26	25.65	**1 : 2.19**

All results in atomic%

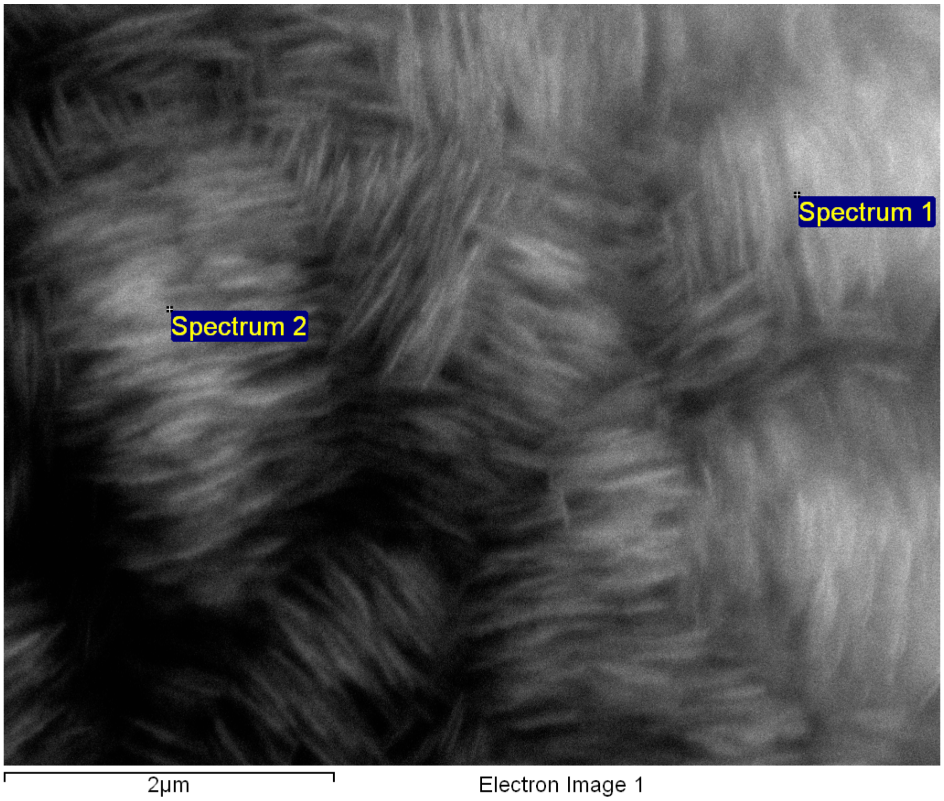

LM6009 st63   FIGURE 1C

Processing option : All elements analysed (Normalised)

Spectrum	In stats.	Si	S	Fe	**Fe:S**
Spectrum 1	Yes	0.52	67.41	32.07	**1 : 2.11**

All results in atomic%

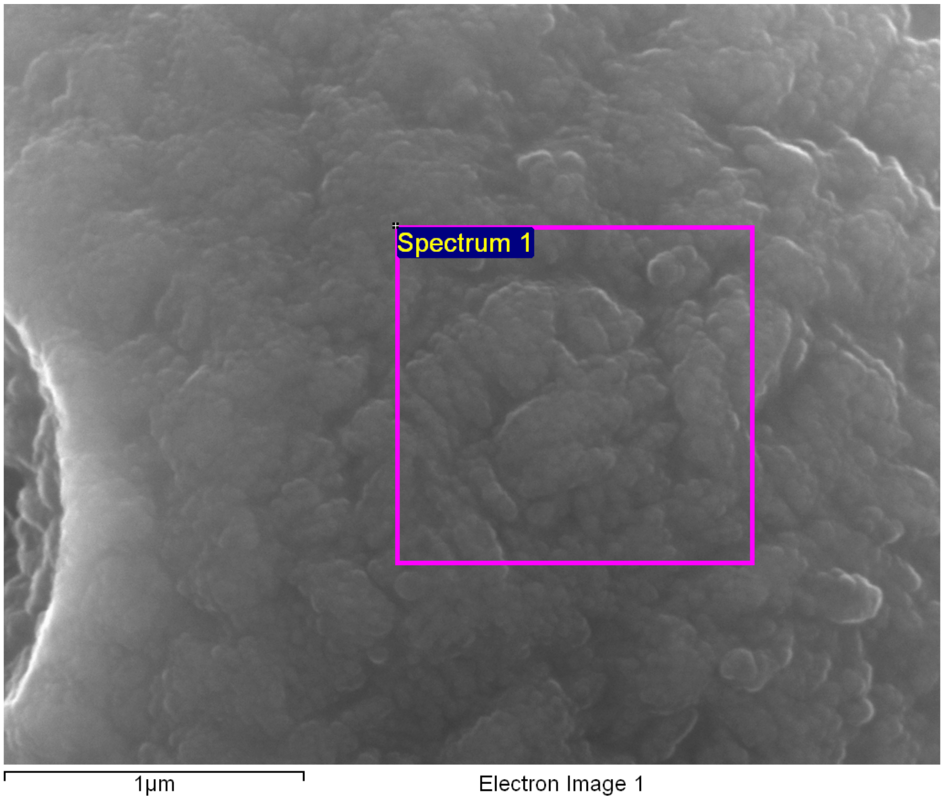

LM6009 st62   FIGURE 1C-insert

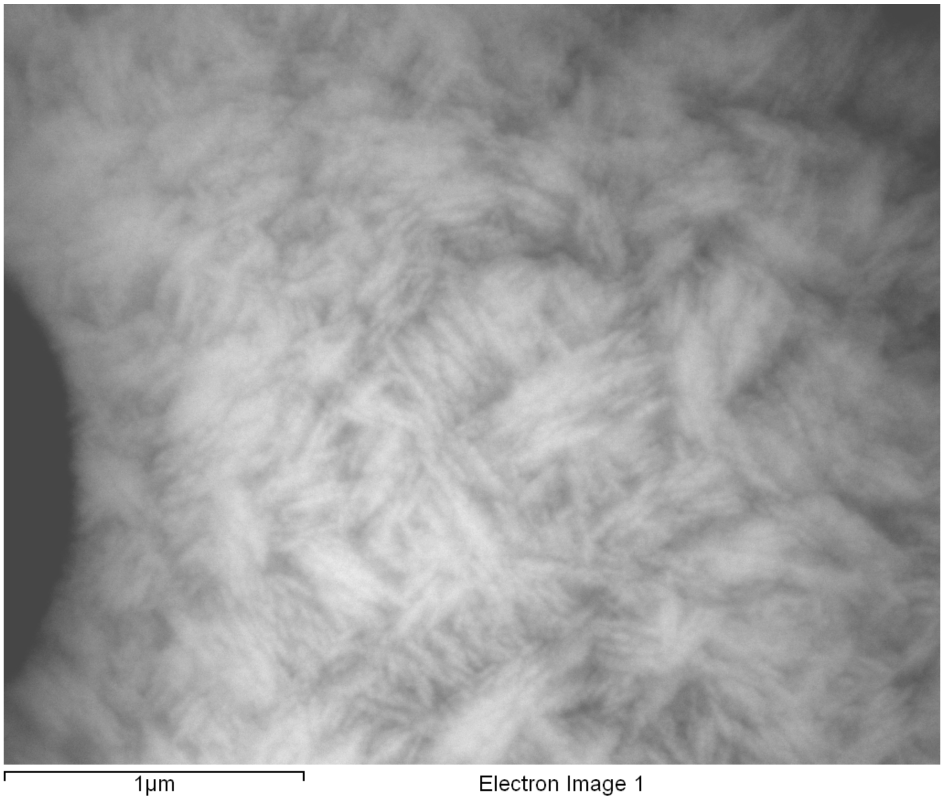

LM6009 st47 FIGURE 1D (see next for BSE of same particle)

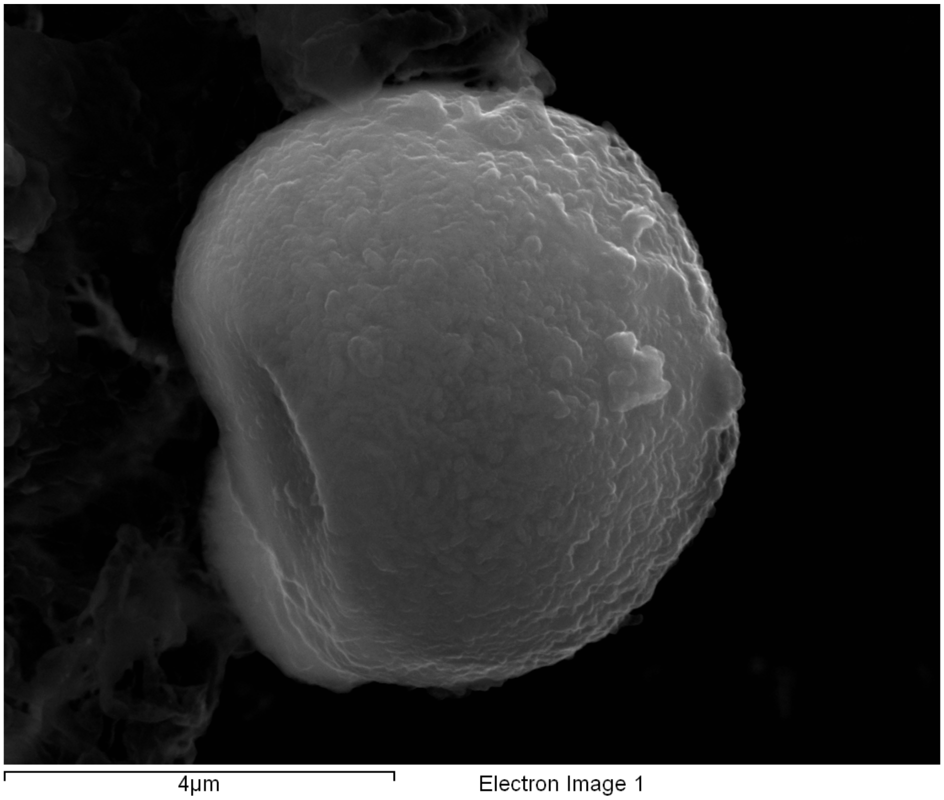

LM6009 st49 FIGURE 1D-insert

Processing option : All elements analysed (Normalised)

Spectrum	In stats.	Si	S	Fe	**Fe:S**
Spectrum 1	Yes	1.16	53.01	45.82	**1 : 1.16**

All results in atomic%

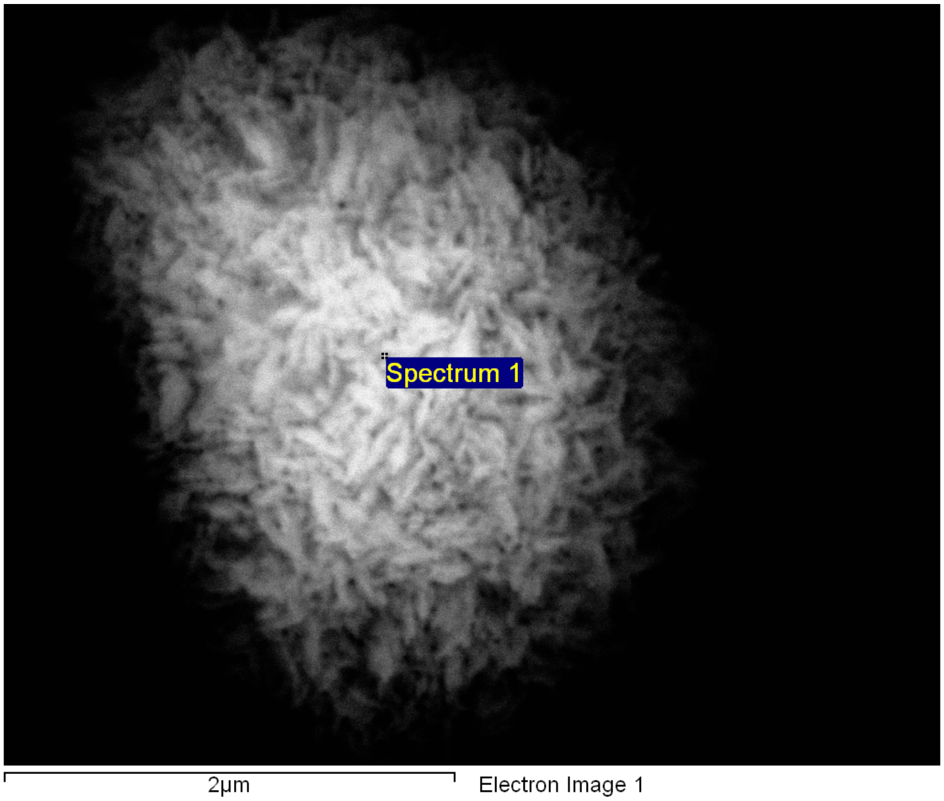

LM6009 st30 FIGURE 1E

Processing option : All elements analysed (Normalised)

Spectrum	In stats.	O	Si	S	Fe	**Fe:S**
Spectrum 1	Yes	75.54	1.76	11.91	10.79	**1 : 1.10**
Spectrum 2	Yes			53.69	46.31	**1 : 1.16**
Spectrum 3	Yes	34.50	1.41	43.02	21.08	**1 : 2.04**
Spectrum 4	Yes	22.10	1.55	42.29	34.06	**1: 1.24**

All results in atomic%

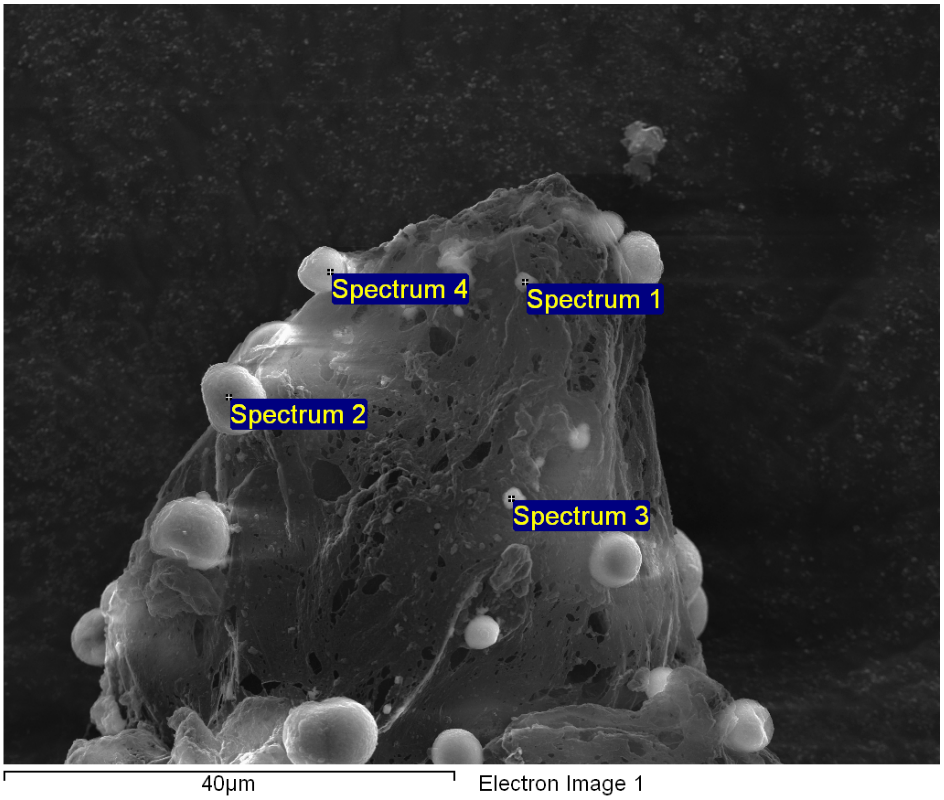

LM6009 st32   FIGURE 1E-insert

All results in atomic%

Spectrum	In stats.	O	Si	S	Fe	Fe:S	
Spectrum 1	Yes		0.66	53.34	46.01	**1 : 1.16**	
Spectrum 2	Yes	12.52	1.52	47.48	38.47	**1 : 1.23**	
Spectrum 3	Yes		1.09	53.48	45.43	**1 : 1.18**	**EDS time 100 seconds**
Spectrum 4	Yes	20.80	2.18	43.58	33.45	**1 : 1.30**	**EDS time 100 seconds**
Spectrum 5	Yes	60.88	1.31	32.48	5.33		
Spectrum 6	Yes	79.62		18.20	2.18		
Spectrum 7	Yes	55.37	2.91	33.39	8.34		

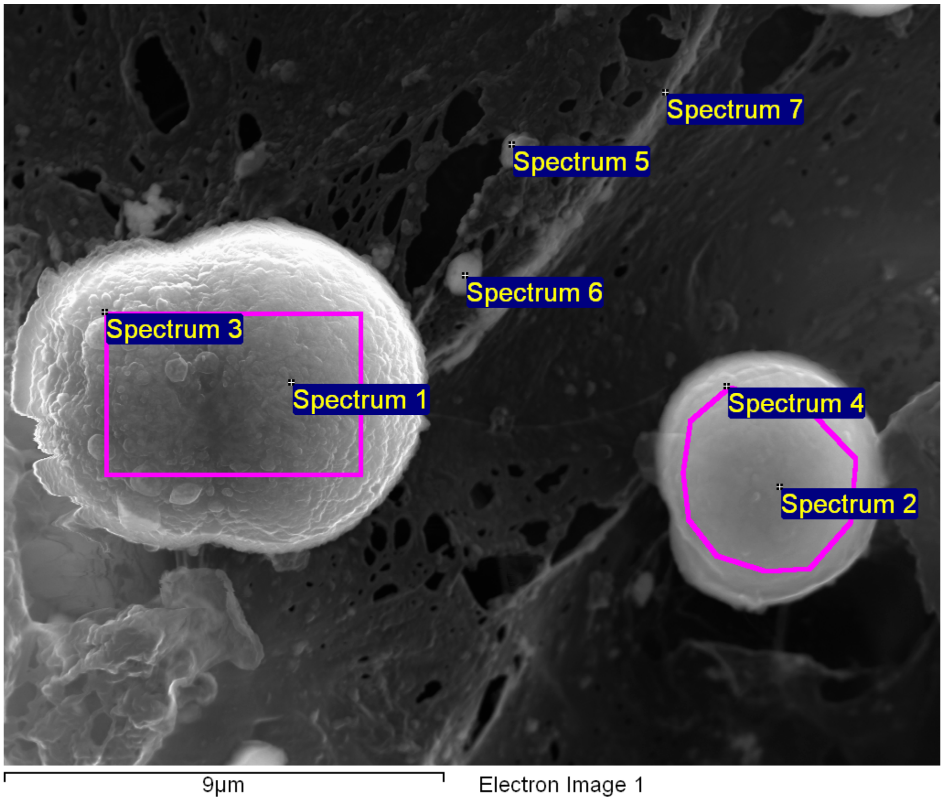

Supplement: Supplementary file 4 — Supplementary Information 4. [file 41598_2024_66006_MOESM4_ESM.pdf]
